# Comparative whole genome DNA methylation profiling across cattle tissues reveals global and tissue-specific methylation patterns

**DOI:** 10.1186/s12915-020-00793-5

**Published:** 2020-07-06

**Authors:** Yang Zhou, Shuli Liu, Yan Hu, Lingzhao Fang, Yahui Gao, Han Xia, Steven G. Schroeder, Benjamin D. Rosen, Erin E. Connor, Cong-jun Li, Ransom L. Baldwin, John B. Cole, Curtis P. Van Tassell, Liguo Yang, Li Ma, George E. Liu

**Affiliations:** 1 Key Laboratory of Agricultural Animal Genetics, Breeding and Reproduction of Ministry of Education & College of Animal Science and Technology, Huazhong Agricultural University, Wuhan, 430070 China; 2 Animal Genomics and Improvement Laboratory, Agricultural Research Service, USDA, Beltsville, MD 20705 USA; 3 National Engineering Laboratory for Animal Breeding, Key Laboratory of Animal Genetics, Breeding and Reproduction of Ministry of Agriculture and Rural Affairs, College of Animal Science and Technology, China Agricultural University, Beijing, 100193 China; 4 MRC Human Genetics Unit, Institute of Genetics and Molecular Medicine, University of Edinburgh, Edinburgh, EH4 2XU UK; 5 Department of Animal and Food Sciences, University of Delaware, Newark, DE 19716 USA; 6 Department of Animal and Avian Sciences, University of Maryland, College Park, MD 20742 USA

**Keywords:** Cattle, Somatic tissues, DNA methylation, Partially methylated domains, Hypomethylated region, WGBS (whole genome bisulfite sequencing)

## Abstract

**Background:**

Efforts to improve animal health, and understand genetic bases for production, may benefit from a comprehensive analysis of animal genomes and epigenomes. Although DNA methylation has been well studied in humans and other model species, its distribution patterns and regulatory impacts in cattle are still largely unknown. Here, we present the largest collection of cattle DNA methylation epigenomic data to date.

**Results:**

Using Holstein cattle, we generated 29 whole genome bisulfite sequencing (WGBS) datasets for 16 tissues, 47 corresponding RNA-seq datasets, and 2 whole genome sequencing datasets. We did read mapping and DNA methylation calling based on two different cattle assemblies, demonstrating the high quality of the long-read-based assembly markedly improved DNA methylation results. We observed large differences across cattle tissues in the methylation patterns of global CpG sites, partially methylated domains (PMDs), hypomethylated regions (HMRs), CG islands (CGIs), and common repeats. We detected that each tissue had a distinct set of PMDs, which showed tissue-specific patterns. Similar to human PMD, cattle PMDs were often linked to a general decrease of gene expression and a decrease in active histone marks and related to long-range chromatin organizations, like topologically associated domains (TADs). We tested a classification of the HMRs based on their distributions relative to transcription start sites (TSSs) and detected tissue-specific TSS-HMRs and genes that showed strong tissue effects. When performing cross-species comparisons of paired genes (two opposite strand genes with their TSS located in the same HMR), we found out they were more consistently co-expressed among human, mouse, sheep, goat, yak, pig, and chicken, but showed lower consistent ratios in more divergent species. We further used these WGBS data to detect 50,023 experimentally supported CGIs across bovine tissues and found that they might function as a guard against C-to-T mutations for TSS-HMRs. Although common repeats were often heavily methylated, some young Bov-A2 repeats were hypomethylated in sperm and could affect the promoter structures by exposing potential transcription factor binding sites.

**Conclusions:**

This study provides a comprehensive resource for bovine epigenomic research and enables new discoveries about DNA methylation and its role in complex traits.

## Background

DNA methylation plays important roles in tissue differentiation and normal developmental processes like gene expression, genomic imprinting, repression of transposable elements, and gametogenesis [1–5]. Many tissue-specific differentially methylated regions (DMRs) were identified and proposed to mediate tissue-specific gene regulatory mechanisms in humans [6]. Earlier studies profiling DNA methylomes in humans and rodents have also shown low methylation near promoters and high methylation in the bodies of active genes [7, 8]. But the relationship between methylation and expression is context-dependent. For example, Varley et al. reported that CpG-rich enhancers in the bodies of expressed genes are actually unmethylated [9].

Partially methylated domains (PMDs) were first discovered in human cell lines and cancers [10]. PMDs were later detected in most mammalian placentas and mouse germline cells [11–14], covering up to 75% of the genomes. As one of the prominent signatures of long-range epigenomic organization, PMDs are large domains of DNA (often greater than 100 kb) which have lower levels of DNA methylation and are associated with gene repression. Early human and mouse analysis identified PMDs as important general, lineage-, and cell type-specific topological features [15]. As Salhab et al. [15] pointed out, changes in PMDs are hallmarks of cell differentiation, with decreased methylation levels and increased heterochromatic histone marks, which are linked to domains of early, middle, and late DNA replication and cell proliferation. However, the patterns and the function impacts of PMDs in cattle are still not known.

CpG sites occur with high frequency in genomic regions called CpG islands (CGIs), which are one of the most widely studied regulatory features. Commonly used cattle CGIs are usually predicted from DNA sequence using computer programs, such as the one downloaded from the University of California Santa Cruz (UCSC) Genome Browser [16]. Although CGIs have critical roles in development and disease, recent studies have shown that such computational annotations are not totally accurate [17]. On the other hand, hypomethylated regions (HMRs, hundreds bp in length) often are located in CGIs and linked to the activation of gene expression; however, they also occur outside of CGIs and function as cell type-specific enhancers [9]. As has been reported [18–20], the formation of HMRs can be due to two possible mechanisms: (1) active transcription and accompanying histone marks such as H3K4me3 prevent the access of DNA methyltransferases and (2) specific protein/DNA complexes, such as CTCF and Sp1, inhibit the methylation machinery in the absence of transcription.

Compared to somatic cells, sperm cells undergo nearly complete reprogramming of DNA methylation and exchange histones by protamine [21–23]. We previously profiled the DNA methylome of cattle sperm through comparison with somatic cells from three bovine tissues (mammary gland, brain, and blood) [24]. Large differences between cattle sperm and somatic cells were observed in the methylation patterns of global CpGs, pericentromeric satellites, and common repeats. Although most of common repeats were heavily methylated in both sperm and somatic cells, we did find some hypomethylated repeats were enriched in gene promoters of sperm cells. Common repeats or transposable elements constitute roughly half of most mammalian genomes [25]. Repression of these common repeats relies on DNA methylation via the piRNA pathway and is essential for the maintenance of genomic stability in the long term and for germ cell function in the short term [26, 27]. In humans, common repeats were found to be heavily methylated—with the notable exclusion of young AluY and AluYa5 elements in human sperm cells [28]. If methylation is lost on certain repressed repeats, germ cell development is arrested in meiosis [29].

Our knowledge of DNA methylation patterns in livestock is still limited when compared to humans and other model species. Some DNA methylation studies were reported with limited tissue types and/or low resolution in cattle, pigs, sheep, and horses [24, 30–46]. For example, we performed comparative analyses of sperm DNA methylomes among human, mouse, and cattle and provided insights into epigenomic evolution and complex traits [47]. To understand the variability of DNA methylation across cattle tissues and its regulation of gene expression, we profiled the cattle DNA methylomes in 16 major tissues using the whole genome bisulfite sequencing (WGBS) method. We investigated the landscapes of the DNA methylome across tissues. We studied differential methylation by comparing them in multiple contexts, including global CpG sites, PMDs, HMRs, CGIs, and common repeats. In line with the Functional Annotation of Animal Genome (FAANG) project [48], this study provides a comprehensive resource for bovine epigenomic research and enables new discoveries about DNA methylation and its role in complex traits.

## Results

### Data generation and quality assessment

We generated 29 WGBS datasets for 16 tissues from 2 Holstein cows and their relatives, including biological replicates whenever possible. These also included 47 corresponding transcriptome datasets for 14 of the 16 tissues and 2 whole genome sequencing datasets (Additional file 2: Table S1A and S1B). Besides 10 published datasets (4 sperm, 2 brain prefrontal cortex, 2 mammary gland, 2 whole blood samples from GSE106538, 24), the other 19 WGBS datasets were newly generated from samples of 2 rumen, 2 lung, 2 *Latissimus dorsi* muscle, 2 adipose, 1 heart, 1 ileum, 1 liver, 1 kidney, 1 spleen, 1 ovary, and 1 uterus collected from the two cows, as well as 2 white blood cell and 2 placental samples from their 4 female relatives. We obtained vast amounts of data, and for each of them, the average unique mapped read count was approximately 150 million (Additional file 2: Table S1). We then uniformly applied Bismark [49] for read mapping and DNA methylation calling, based on two cattle assemblies, i.e., short-read-based UMD3.1.1 [50] versus long-read-based ARS-UCD1.2 [51]. Although the differences of mapping rates and global methylation levels were small between two different assemblies (Additional file 2: Table S2), many DNA methylation-level peaks and valleys did change their locations and magnitudes, especially when they were near chromosome ends (i.e., pericentromeres or telomeres) or sudden drops of the DNA methylation level (Additional file 1: Figure S1 and S2). Considering the high quality of the long-read assembly, such as the better context identification and future applicability, we focused all following analyses using the long-read-based assembly ARS-UCD1.2. We used Bismark [49] to identify genome-wide methylated cytosines, which gave a median coverage of 16 × (coverage) per sample (range from 11.84 to 24.47×) (Additional file 2: Table S1). To remove the SNP artifacts from the subsequent analyses, we filtered away all SNPs detected in the whole genome sequence of the same individual (Additional file 2: Table S1C).

### Dynamic DNA methylation for tissue-specific development in cattle

In terms of the global CpG methylation level of the cattle genome DNA, we obtained consistent results as compared to human [52], mouse [53], and other species. Most somatic tissue DNA samples in cattle had average methylation levels of 70~80%, while the placental genomic DNA was 48% (the least) methylated, as compared to the sperm DNA which was ~ 78% (the most) methylated (Additional file 2: Table S1A). Bisulfite conversion rates estimated by unmethylated lambda DNA controls supported that we faithfully captured patterns of genomic DNA methylation in these samples (Additional file 2: Table S1A). Moreover, we detected low non-CG methylation in the non-brain somatic tissues and sperm cells (0.2–0.8%), in contrast to a higher non-CG methylation level in the brain samples (1.2–1.3%). The latter was consistent with previous studies in human and other species [54].

**Fig. 1:**
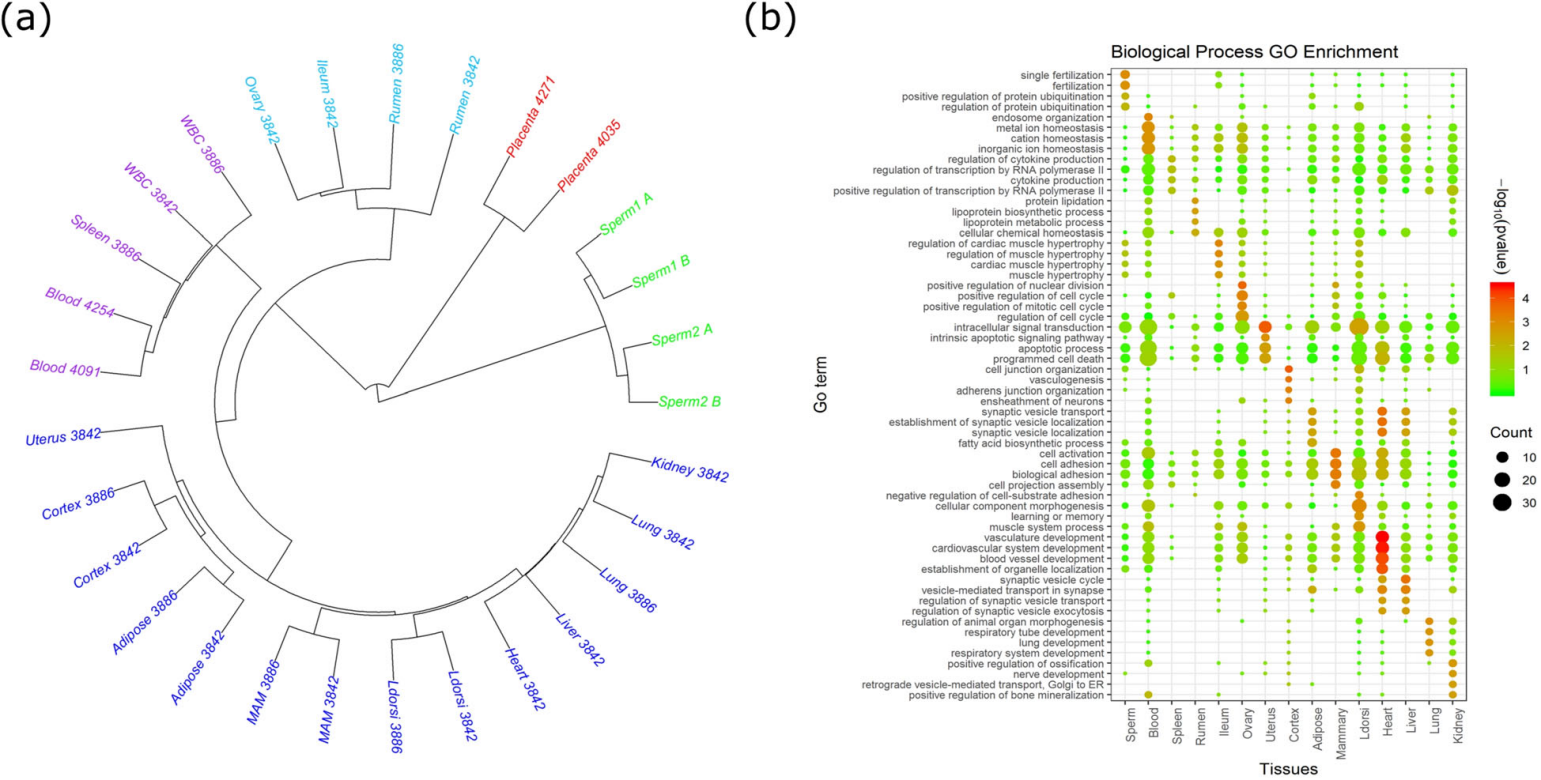
Analyses of DNA methylation for the tissue-specific development in cattle. **a** Cluster analysis of the samples using the average methylation level of 500-bp windows. **b** Gene ontology analysis for the genes overlapped with tissue-specific DMRs

We performed a hierarchical clustering of these tissues based on weighted methylation levels of 500-bp windows (Fig. 1a). As expected, they were organized into three main clusters: sperm cells (cluster 1), placenta (cluster 2), and somatic tissues (cluster 3). These results confirmed the consistent results between the biological replicates and reinforced potential methylation differences among different somatic tissues, placentas, and sperm cells (Fig. 1a). Additionally, independent principal component analysis (PCA) also confirmed this clustering (Additional file 1: Figure S3a). PC1 successfully separated samples into 3 clusters (sperm, placentas, and somatic tissues), which explained most (44.90%) of the variances, while PC2 separated sperm and placentas from somatic tissues. PC3 separated the blood/white blood cell/spleen from the rest and PC4 separated the rumen from all other samples.

We compared the methylation profiles between pairs of samples at a global CpG level. As expected, the correlations between samples within the same tissue type or clusters were high (*r* > 0.5) (Additional file 1: Figure S4). The correlations between methylation of different tissue types or clusters were lower, especially those between sperm cells and somatic tissues were the lowest (*r* = 0.22 to 0.24) (Additional file 1: Figure S4).

Focusing on cluster 3 of Fig. 1a, we saw much more conserved methylation patterns when they were compared to the sperm cells and placenta in all 3 analyses (clustering, PCA, and correlation in Fig. 1a, S3, and S4, respectively). The somatic tissues in cluster 3 can be further divided into 3 branches with stronger correlations among their DNA methylome levels (Fig. 1a). It is noted that samples from the same germ layers and/or the same biological systems were clustered together (e.g., heart and muscle, spleen and blood, and adipose tissue and mammary gland). This was in agreement with the common notion about the importance of methylation in tissue-specific development. Moreover, several tissues showed specific methylation patterns in cattle. For example, the blood cluster (cluster b) and the rumen were separately divided by PC3 (6.54%) and PC4 (4.39%) from other tissues (Additional file 1: Figure S3b). Interestingly, we also found increased correlation coefficients of methylation levels toward the sperm cells and placenta especially for the cortex and rumen, respectively (Additional file 1: Figure S5). Using 10-kb, non-overlapping windows, we analyzed the conserved and variable methylation regions on the cattle genome across all samples, based on the standard deviations of DNA methylation levels. We defined the lowest 1% tails as methylation conserved regions, while the highest 1% tails as methylation variable regions (Additional file 1: Figure S6). We first divided methylation conserved regions into 2 parts: hypermethylation conserved regions and hypomethylation conserved regions. We found that genic regions were most enriched in hypermethylation conserved regions (Additional file 1: Figure S7). The genes located in the hypermethylation conserved regions were highly enriched in the DNA damage and repair biological processes (Additional file 1: Figure S8a). In the hypomethylation conserved regions, some of important functional genomic features including promoters, eCpG islands, and tRNA genes were highly enriched (Additional file 1: Figure S9). But no significant enriched GO term was found. On the other hand, we found that methylation variable regions were enriched in the promoters and eCGIs (Additional file 1: Figure S10). No significantly enriched GO term was found when we did the GO analysis using all the genes overlapped with the methylation variable regions (Additional file 1: Figure S11). We then separated methylation variable regions into 2 parts according to the sperm methylation level: sperm hypermethylation variable regions and sperm hypomethylation variable regions. We found that the genes located in the sperm hypomethylation variable regions (the methylation variable regions that showed hypomethylation in sperm) showed high enrichment in the meiotic cell cycle process, cell division, and spermatogenesis (Additional file 1: Figure S8b). The genes located in the sperm hypermethylation variable regions (the methylation variable regions that showed hypermethylation in sperm) showed high enrichment in the response to hormone, multi-multicellular organism process (Additional file 1: Figure S8c). Additionally, methylation variable regions in our heatmap analysis (Additional file 1: Figure S12) successfully separated the samples into three similar groups (placenta, sperm, and other somatic tissues), as we observed in our hierarchical clustering based on weighted methylation levels (top 500-bp tails) (Fig. 1a). Therefore, as described above, our results showed that the methylation conserved regions played important roles for the basic, key bioprocesses, while the methylation variable regions were associated with tissue-specific activities.

We identified 5.85 million differentially methylated cytosines (DMCs, methylation differences > 0.3, FDR < 0.05, with 10× in depth) that distributed in 215,984 differentially methylated regions (DMRs, methylation differences > 0.3, FDR < 0.05, supported by at least 5 DMCs in the same direction) between any one tissue sample against tissues in all other groups throughout the cattle genome (Additional file 2: Table S3). As the placenta was lowly methylated, we detected the largest number of DMCs and DMRs covering half of the genome. Among somatic tissues and sperm cells, we identified the tissue-specific DMRs and found the hypomethylated genes overlapped with the tissue-specific DMRs were enriched in the tissue-specific development GO terms, for example, fertilization for the sperm, positive regulation of the nuclear division for the ovary, and vasculature development for the heart (Fig. 1b). These results indicated large differences between tissue methylomes were likely related to tissue-specific development and function.

### PMD

As described before [24], we applied an HMM model to detect partially methylated domains (PMDs) using 10-kb windows (Additional file 2: Table S4), whose lengths are usually over hundreds of kilobases. Previous studies have recognized that the placenta has a similar epigenetic landscape as cancer cells have, which are characterized by a widespread hypomethylation in human and mice [13]. Here, we found that the cattle placenta contributed 80% (~ 1.2 Gb) of all PMDs in length and covered most of the PMDs detected in the other tissues. Cattle placenta PMDs covered 40.83 and 43.74% of the cattle genomes, respectively. On the other hand, PMD only occupied 30.29% of the human placenta genome, agreeing with previous estimates [13]. We further determined genes located in the shared and lineage-specific placenta PMDs between cattle and human (Additional file 2: Table S5). GO terms with significant enrichment for genes shared in PMDs included chemical synaptic transmission, adhesion junction organization, ion transmembrane transport, and nervous system development. Genes for human-specific PMDs showed one marginally significant enrichment for anterior/posterior pattern specification, while cattle-specific PMDs showed one significant enrichment for steroid hormone-mediated signaling pathway (Additional file 2: Table S6).

**Fig. 2:**
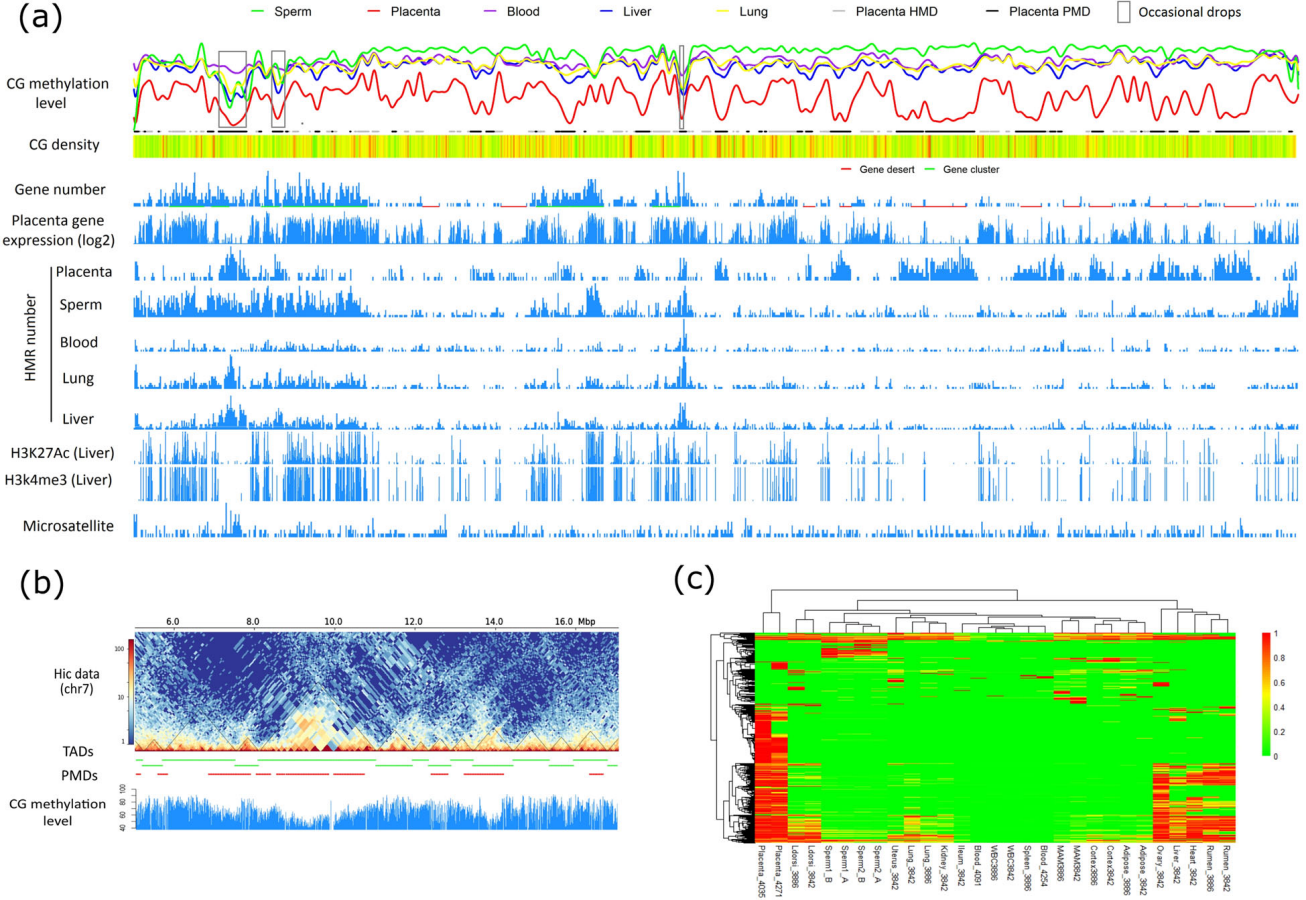
DNA methylation landscapes of PMDs in various cattle tissues. **a** Distribution analysis of the PMD using chr7 as an example with different genome tracks, including from the top to bottom: CG methylation level; CG density; gene number; placenta gene expression (in log2 scale); HMR numbers in the placenta, sperm, blood, lung, and liver; H3K27Ac (liver) and H3K4me3 (liver). Three DNA methylation-level drops are labeled out with rectangles. **b** Comparison of the location between PMDs and TADs. **c** Cluster analysis of cattle samples by the PMD. The PMDs were merged into PMD regions for all the samples. We calculated the ratio of the PMD of each sample to the merged PMD regions, which was then used for the cluster analysis

After merging all cattle tissue PMDs, we found that over half (~ 1.45 Gb) of the whole cattle genome were covered by PMDs in at least one sample. However, PMD mostly existed in the gene desert (gene poor region) with low CG density and often lack of the actively histone modifications, including H3K27Ac and H3K4me3 (Fig. 2a, Additional file 2: Table S4). Overlapping with the Hi-C contact maps revealed that TADs were often associated with PMDs on the cattle genome (Fig. 2b). We did find several PMDs that overlapped with parts of the genes or gene cluster regions. But most of PMDs often showed a genome-wide inhibition of gene expression in all samples (Additional file 1: Figure S13). Moreover, we identified highly methylation domains (HMDs) using the HMM strategy in the placenta. Placenta HMDs covered 27.58% of the cattle genome and were enriched for the gene-related features, including genic regions, promoters, experimentally supported CGIs (eCGIs, presented in the later part of the “Results” section) (Additional file 1: Figure S14), and 7057 (54.80%) RefGenes. In PMDs, the methylation patterns of the genes and the CGI were almost indistinguishable from the background because of their low methylation (Additional file 1: Figure S15). But the genes in HMDs were significantly enriched in the basic biological processes, including intracellular protein transport, DNA repair, apoptotic process, endocytosis, and cell division (Additional file 2: Table S7). This may help to explain how the placenta functions normally even with a large percentage of PMDs. Genes in placenta PMDs were significantly enriched in the following GO terms: homophilic cell adhesion via plasma membrane adhesion molecules, gamma-aminobutyric acid signaling pathway, pituitary gland development, chemical synaptic transmission, and anterior/posterior pattern specification (Additional file 2: Table S8).

We further examined whether the cattle PMDs could be used as markers for different tissues. We found the replicates for the same tissues were successfully clustered together using the PMDs (Fig. 2c). Most of the samples, except the placenta, had small proportions of PMDs. Within them, we found the rumen, heart, liver, and ovary had more PMDs than others. It is of note that the blood and its related sample (spleen) showed the least numbers and shortest lengths of PMDs (Additional file 2: Table S4). Through a visual examination, we detected multiple methylation-level drops that commonly appeared in the non-blood samples at the chromosome level (Fig. 2a labeled with narrow squares, and Additional file 2: Table S9). We then identified those DNA methylation-level drop regions on the chromosome using a PMD-cluster strategy. Finally, we identified 16 non-blood DNA methylation-level drops ranging from 0.3~1.8 Mb in length, which were distributed on 13 different chromosomes (Additional file 2: Table S9). Five of them overlapped with gene cluster regions with gene family loci related to immunity, histone, olfactory receptor, pregnancy-associated glycoprotein, and protocadherin. The others were enriched in satellite. Since all bovine autosomes are acrocentric and pericentromeres are not well defined in the cattle genome, the first 3 Mb of them could be considered as potential pericentromeres. Based on this criterion, 9/16 of them were located in pericentromeres (Additional file 2: Table S9).

### TSS-HMR as an important indicator for gene expression

**Fig. 3:**
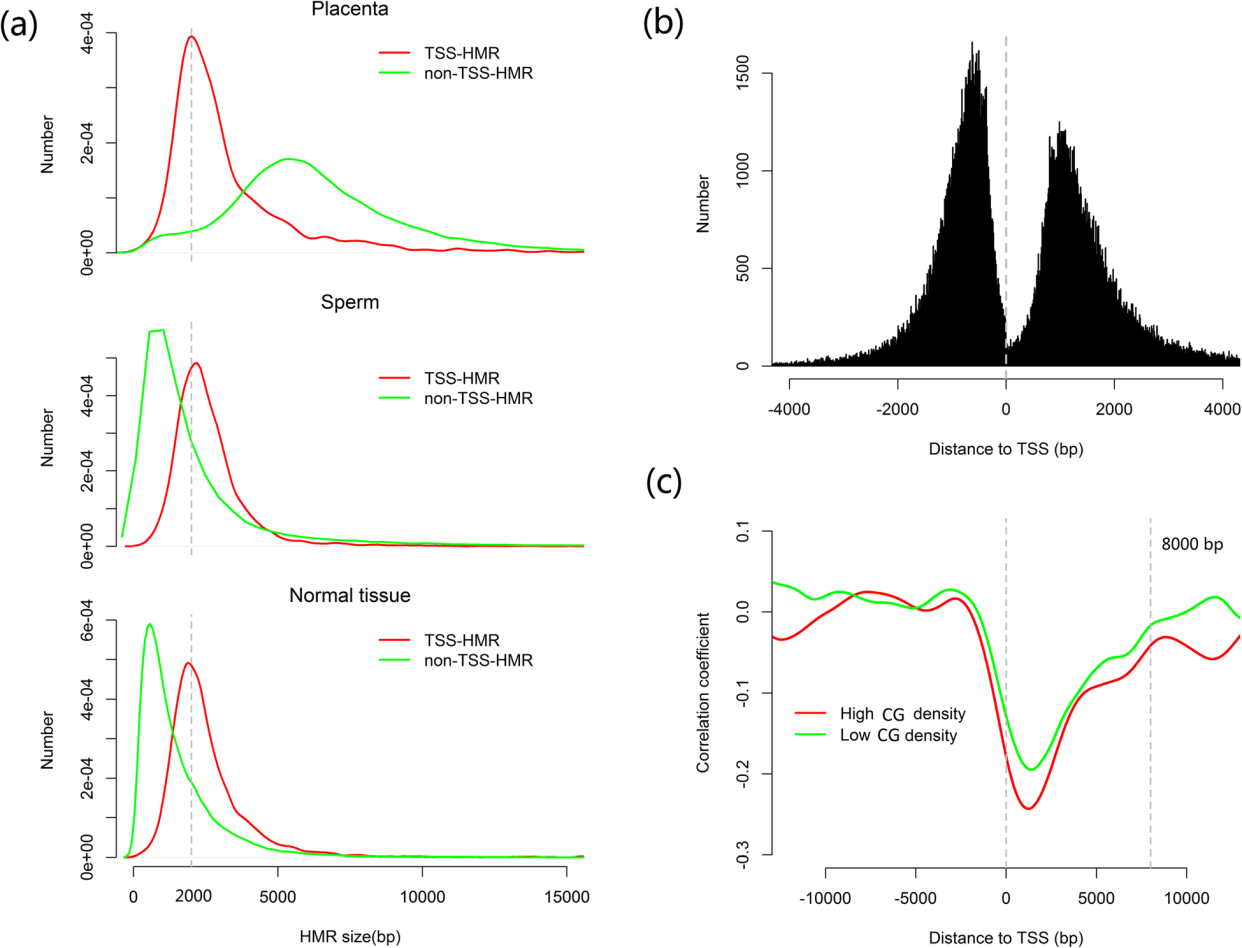
TSS-HMR as an important indicator for gene expression. **a** Comparison of the size distributions of different kinds of HMR between the samples in three clusters. **b** Analysis of the flanking boundaries of the TSS-HMR. **c** Correlation analysis of the DMRs with gene expression across the gene body region

We also applied an HMM module to detect the hypomethylated regions (HMRs) for each sample, as described previously [24]. In total, we found that the HMR covered 847 Mb of the cattle genome. However, the HMRs showed large differences in both location and size among the different clusters (Fig. 2a and Additional file 1: Figure S16). In somatic tissues and sperm, HMRs were highly enriched in the promoters, eCGIs, tRNAs, and satellites, while the placenta samples showed the opposite trend (Additional file 1: Figure S17). We divided the HMRs into two types according to their overlapping with TSS or not: TSS-HMR and non-TSS-HMR. The peak size of non-TSS-HMR for the placenta was around 5000 bp and those for sperm and normal tissues were much smaller in size (around 500 bp) (Fig. 3a). However, interestingly, the peak sizes of TSS-HMR were highly consistent, centering around 2000 bp among all samples (Fig. 3a). This might indicate the importance of the TSS-HMR throughout tissue development.

We calculated and plotted the distances from the two HMR boundaries to the nearest TSS. Of note, we found that the center of the HMR was usually located in the downstream of the TSS (Fig. 3b). We examined the relationship between methylation and transcription, using a correlation analysis between the methylation levels of intragenic DMRs and the expression of the closest genes based on 20 methylome and transcriptome data derived from the same samples in cluster 3. As expected, high methylation in DMRs had a negative correlation with gene expression, and this negative correlation grew stronger around the transcription start site (Fig. 3c). The strong negative correlation was not only in the gene promoters, but also extended downstream of the promoter up to 8 kb away (Fig. 3c). This analysis shows that transcription is strongly associated with intragenic DMRs in the tissues we examined, in line with the similar observations in human methylomes [52]. Additionally, as expected, we found the TSS-HMR with high CG density showed a stronger negative correlation with gene expression than TSS-HMR with low CG density did (Fig. 3c).

We then counted commonly and differentially methylated TSS-HMR for the homologous genes between cattle and human for either the liver or the kidney, as we did previously for sperm [47]. Over 90% genes with TSS-HMR were conserved (i.e., commonly methylated, either highly or lowly methylated) between cattle and human: 91.70% for the liver or 94.03% for the kidney. These liver and kidney genes with conserved methylated TSS-HMR were involved in basic biological processes, like RNA processing, protein folding, and cell cycle (Additional file 1: Figure S18). On the other hand, we did find 69 homologous genes with differentially methylated TSS-HMR (Additional file 2: Table S10). They included some lipid-related genes (e.g., *CYP11A1*, *PLD6*, *MGLL*, *CYP39A1*, *RAB7A*), which were hypomethylated in human. Human liver and kidney also shared 8 genes with hypomethylated TSS-HMR. However, their mechanisms are not well understood and require future investigations.

### Classification of the TSS-HMR: tissue-specific TSS-HMR for cattle gene expression

**Fig. 4:**
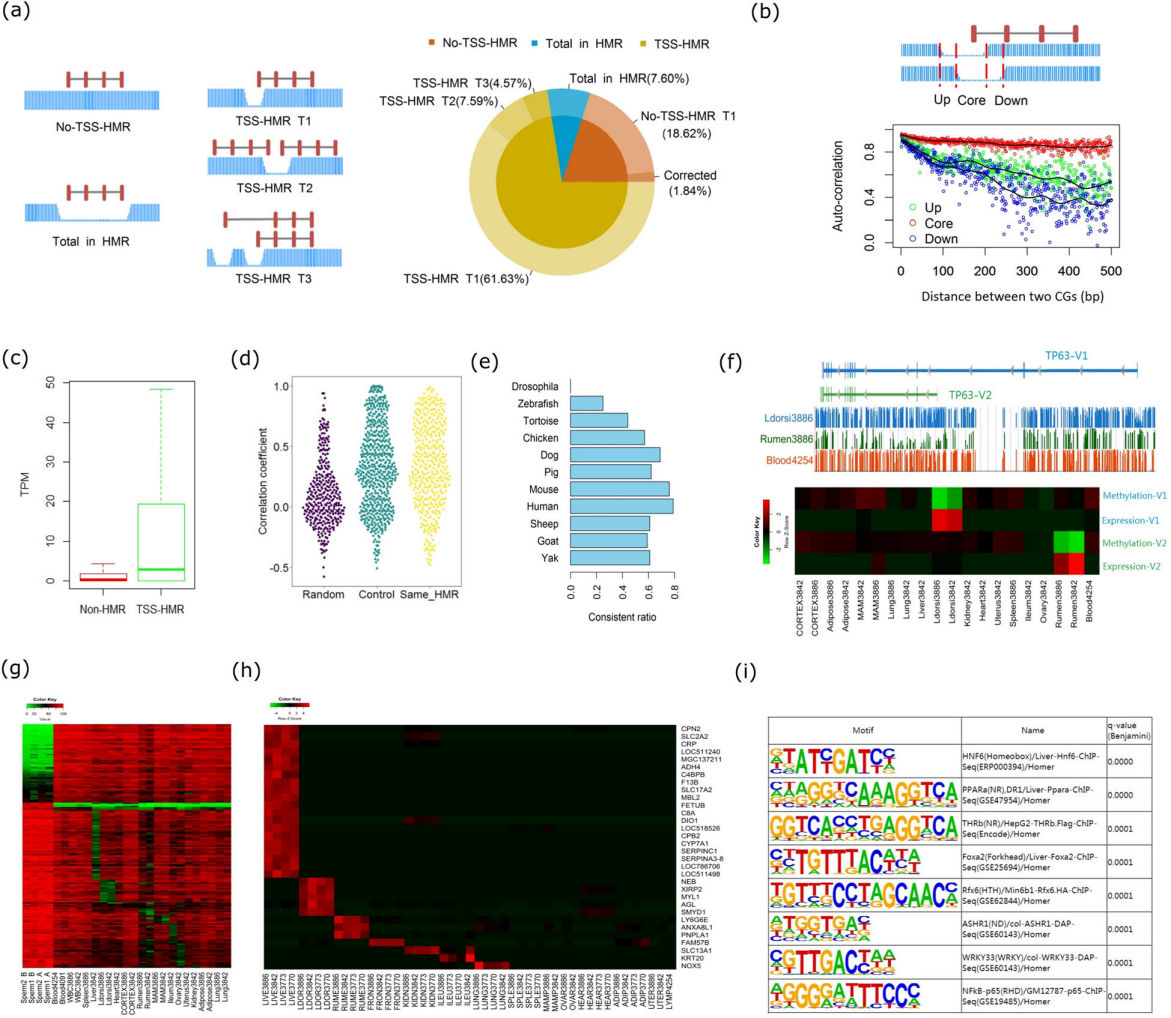
Classifications of TSS-HMR and their tissue specificity. **a** Classification of the 5 types of the TSS-HMR: (1) no-TSS-HMR; (2) total in HMR; (3) TSS-HMR T1; (4) TSS-HMR T2; (5) TSS-HMR T3. Please refer to the main text for details. **b** Comparison of the methylation-level correlation of the CG with different distances between the TSS-HMR core region and two flank regions. **c** Boxplots of the gene expression for the genes with TSS-HMR or not. **d** Correlation analysis of the expression of the gene pairs with TSS located in the same HMR. Random: same number of genes pairs randomly chosen from all genes; control: same number of gene pairs randomly chosen from all genes with the same distance of the two TSS; Same_HMR: the gene pairs with TSS located in the same HMR. **e** The consistent ratio of gene pairs with TSS located in the same HMR of cattle in the other species, we defined the nearest two different strands genes as gene pairs. **f** The example of *TP63*: its tissue-specific expression of different transcripts from the two different TSS were regulated by the two tissue-specific TSS-HMRs. **g** Heatmap of the methylation level of tissue-specific TSS-HMR. **h** Heatmap of the expression levels of the genes showing tissue-specific high expression and tissue-specific TSS-HMR. **i** Transcription factor binding motif enrichment analyses of the tissue-specific TSS-HMR using the liver as an example

To better understand the TSS-HMR, we classified genes into 5 groups according to their promoter location relative to the TSS-HMR by integrating the WGBS data of 27 diverse samples (except placentas as their methylation levels were lower) and their corresponding RNA-seq data. The 5 gene groups were as follows: (1) no-TSS-HMR: the gene with its TSS not located in the HMR in all samples; (2) total in HMR: the gene totally located in the HMR in at least one sample; (3) TSS-HMR T1: only one gene with its TSS located in the HMR in at least one sample; (4) TSS-HMR T2: two opposite strand genes with their TSS located in the same HMR; and (5) TSS-HMR T3: two or multiple transcripts of one gene with TSSs located in different HMRs (Fig. 4a). According to this classification, we identified 20.46% of the annotated coding genes (NCBI) without TSS in all the samples. To avoid the possible incomplete gene annotation, we fine-mapped TSS for 1.84% of genes using our RNA-seq data. Most (81.4%) of annotated coding genes were classified as TSS-HMR genes, including TSS-HMR T1 (61.63%), TSS-HMR T2 (7.59%), and TSS-HMR T3 (4.57%). The genes (7.60%) totally within HMR were short in length (average length = 2551 bp; medium length = 1726 bp) and enriched in G-protein-coupled receptor signaling pathway, sensory perception of smell, and nucleosome assembly (FDR < 0.01) (Additional file 2: Table S11).

As HMR boundaries varied among different tissues, we then investigated its core and flanking regions. Compared to the two upstream and downstream regions of the HMR, the core regions (shared by the TSS-HMR in all samples) had much higher CG density and were more conserved in terms of the methylation level among different tissues (Additional file 1: Figure S19). The correlation efficient values between the methylation levels of adjacent CGs were kept stably high (> 0.8) even for long distance in the HMR core region while those of the HMR flank regions decreased more rapidly (Fig. 4b). Thus, to study the relationship between methylation and gene expression, we focused on the core region. As expected, the expression of the genes that classified as non-TSS-HMR were mostly suppressed (Fig. 4c).

By performing correlation analyses between the paired genes’ expressions, we found the paired genes (twin-genes) within TSS-HMR T2 would have greater chances of being co-expressed (Fig. 4d). Moreover, we examined the existence of those paired genes in other species. The paired genes were more consistently co-expressed among different mammals and chicken but showed lower consistent ratios in other species, including tortoise, zebrafish, and *Drosophila* (Fig. 4e). For example, we performed similar TSS and HMR analyses using human liver and kidney WGBS datasets. We found 783 such gene pairs in human and 514 (65.65% = 514/738, Additional file 2: Table S12) of them with their TSS overlapped with human HMRs. Hence, this cross-species comparison between cattle and other species revealed the important roles of epigenome evolution in mammals and chicken.

As for genes within the TSS-HMR T3, they have a possibility to be regulated by tissue-specific methylation of TSS-HMR. For an example, the *TP63* gene could be expressed from two different TSSs and their expressions were tissue-specifically regulated by the methylation levels of these two TSS in LD muscle and rumen, respectively (Fig. 4f). We recovered 122,867 cases, involving 4123 genes with different TSS-HMRs in at least two samples and 171 tissue-specific TSS-HMRs (Fig. 4g). We also identified 3207 genes contained by 12 modules that specifically highly expressed in 12 different tissues by performing a weighted gene co-expression network analysis (WGCNA) (Additional file 1: Figure S20). Those genes were enriched highly in GO terms related to the special tissue functions (Additional file 2: Table S13). Combining with the gene expression using the RNA-seq data, we found tissue-specific HMRs for 32 genes were highly correlated with their expression (Fig. 4h). For example, our results showed that liver-specific TSS-HMR for the following genes: *CPN2*, *SLC2A2*, *CRP*, *LOC511240*, *MGC137211*, *ADH4*, *C4BPB*, *F13B*, *SLC17A2*, *MBL2*, *FETUB*, *C8A*, *DIO1*, *LOC518526*, *CPB2*, *CYP7A1*, *SERPINC1*, *SERPINA3-8*, *LOC786706*, and *LOC511498*. After filtering out 4 predicted LOC genes, we queried the left 16 genes against human GTEx portal [55] and cattle gene atlas [56]. Except for 3 genes, all other 13 genes were uniquely express in liver tissues of both human and cattle. *MGC137211* and *SERPINA3-8* were still uniquely expressed in the cattle liver, but they were not found in the human genome. On the contrary, *DIO1* was not found in the cattle genome, and its expression in human thymus was higher than that in human liver. In summary, these cross-species comparisons revealed conserved tissue-specific gene expression were associated with conserved tissue-specific TSS-HMRs. This observation generally agreed with a recent report that conserved tissue-specific transcriptions across species could be more often explained by conserved tissue-specific DMRs [57]. Finally, we found that the tissue-specific TSS-HMRs were strongly enriched for putative binding sites of transcription factors which are known to have tissue-specific function (Fig. 4i). For example, we detected *HNF6*, *PPARA*, and *Foxa2*, which are liver-specific transcription factors and further confirm our above speculations.

### eCGI as a guard to avoid C/T mutation for the TSS-HMR core region

**Fig. 5:**
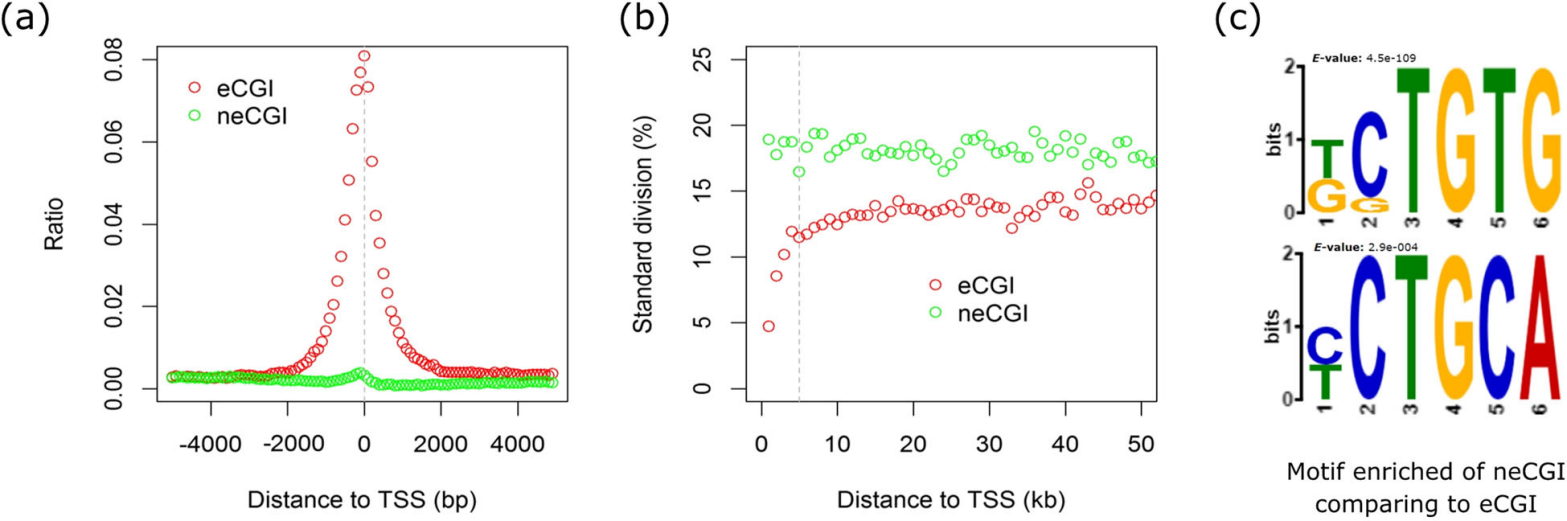
Comparison between the eCGI and neCGI. **a** eCGI was enriched around the TSS. **b** The eCGI was lowly methylated and conserved among different tissues (samples), shown as the standard division of methylation levels among the different samples diminished as their distances to the TSS decreased. **c** Motif enrichment analyses of the neCGI as compared to the eCGI

Previous human studies have shown that the computational annotations of CGI (cCGI) suffer from inaccuracies [17]. Here, we totally identified 50,023 experimentally supported CGIs (eCGIs) at a single-base resolution using 27 whole genome bisulfite sequencing data (not including the two placentas) in cattle (Additional file 1: Figure S21, Additional file 1: Figure S22a, and Additional file 2: Table S14). The neCGI (cCGI not overlapped with eCGI) almost lost and even showed an opposite methylation patterns as compared to those of the eCGI (Additional file 1: Figure S22b). More importantly, we found that the genomic distributions of these eCGIs across chromosomes correlated more strongly with gene contents than with chromosome lengths (Additional file 1: Figure S23). This suggested that these hypomethylated regions might contain regulatory elements for gene expression. Furthermore, eCGI was highly enriched around the TSS as compared to the neCGI, which was actually highly enriched in the telomere of the chromosome (Fig. 5a, Additional file 1: Figure S24). We found eCGI overlapped with 10,503 (62.51%) the TSS-HMR core region, which is consistent with the previous notion of the importance of CGI in the regulation of gene expression [58]. Earlier results also showed that the high methylation of the cytosine within CGI usually leads to C/T mutations more easily [59]. We found that the eCGI was not only kept low in methylation but also its methylation level was more conserved among different tissues (samples) (Fig. 5b). We checked the distribution of the C/T heterozygote (including C/T, G/A) for the eCGI in the two individuals using their genome sequencing data. The C/T heterozygote rate was lower for all eCGI or the eCGI in the TSS-HMR core region (Additional file 1: Figure S25). We searched for motifs enriched in the neCGI as compared to eCGI. As expected, we found more motifs of TG or GA, which were possibly the results of the mutation from CG to TG (Fig. 5c). This provided more reasons for the low methylation level in the eCGI, which might actually protect sequence from C/T mutation in the TSS-HMR.

### Common repeats may regulate gene expression via differential DNA methylation of the newly introduced transcription factor binding sites (TFBS)

**Fig. 6:**
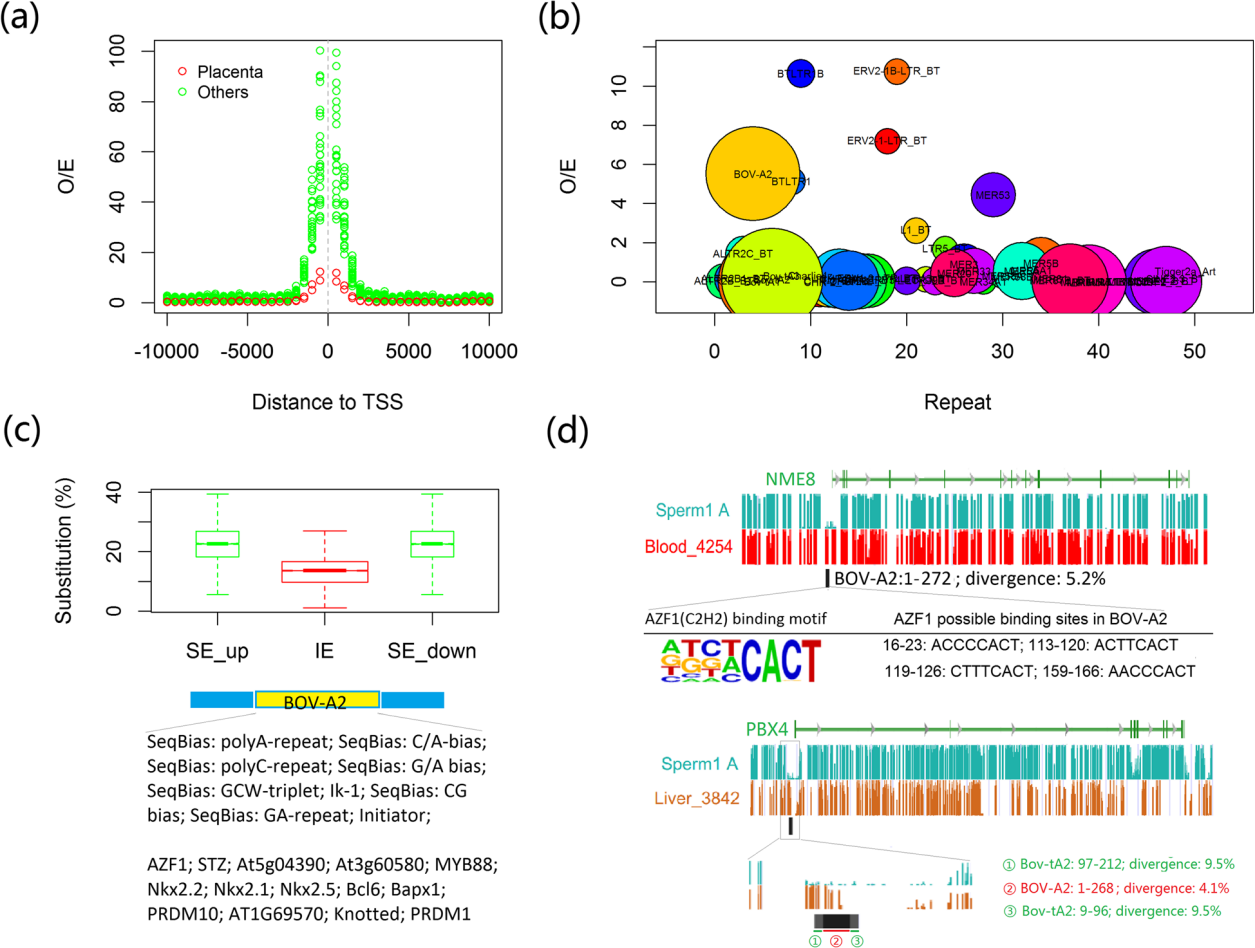
Analysis of hypomethylated repeats. **a** Hypomethylated repeats were enriched around the TSS for different samples. **b** Analyses of the repeats located in the TSS-HMR; observed/expected ratio of the repeats (*y*-axis) plotted for each repeat subclass; the size: the number of the repeats located in the TSS-HMR. **c** Analysis of young Bov-A2 element insertions; SE_up: the upstream part of ancient repeats, SE_down: the downstream part of ancient repeats. IE, the young Bov-A2 elements, which inserted more recently, thus split ancient repeats. The *y*-axis represents the sequence divergences, thus age of repeats. BOV-A2Bov-A2. **d***NME8* and *PBX4* as two examples for the Bov-A2 insertion in the tissues with different TSS-HMRs

Most of the common repeats, especially retrotransposons, showed high methylation levels, which repress their transcriptions (Additional file 1: Figure S27). But there are still some repeat elements hypomethylated (≤ 3.3%), which are highly enriched in the regions around the TSS (2000 bp) (Fig. 6a). We plotted their observed/expected ratios for each repeat subclass (Fig. 6b). We checked whether common repeats would bring special sequences such as transcription factor binding sites (TFBS) to the TSS-HMR. Because repeat elements could be broken into short pieces because of multiple rounds of insertions, we focused on the full-length elements (integrity > 80%) located in the TSS-HMR. In total, we found 4389 elements that dispersed in the TSS-HMR (Additional file 1: Figure S27). We searched for the specific sequences or motifs enriched in the different elements as compared to all TSS-HMR sequences. We observed that only Bov-A2 elements are enriched for multiple known Zinc-finger-related transcription factor binding motifs (Fig. 6c). As an example, we showed the results for a subset of young Bov-A2, which recently inserted into and split ancient common repeats (Fig. 6c). But we did not find any significant GO term for the genes containing Bov-A2 in the TSS-HMR. Interestingly, we detected several hypomethylated Bov-A2 around the TSS-HMR in sperm cells. For example, *NME8*, one known gene related to sperm function, containing one Bov-A2 element insertion with 4 AZF1 binding motifs in its TSS-HMR, was especially hypomethylated in the sperm samples (Fig. 6d). We also found one young Bov-A2 element embedded in an old Bov-tA2 in the promoter region of the *PBX4* (pre-B cell leukemia homeobox 4) gene, which encodes a member of the pre-B cell leukemia transcription factor family. Again, we detected low methylation of this TSS-HMR only in the sperm samples (Fig. 6d).

## Discussion

Using WGBS, we generated one of the first large-scale, single-nucleotide resolution cattle somatic tissue methylomes. Cattle-unique tissue-like rumen was also reported for the first time. The global CG methylation levels detected ranged from 72.8 to 78.1% among our cattle samples, which were similar to those in other mammalian species like humans (~ 70%) [21] and what we reported previously [24]. Our genome-wide cattle methylomes confirmed existing knowledge that DNA methylation is important for gene expression and plays a critical role in tissue-specific processes [5, 60]. In promoter regions, DNA methylation is associated with transcriptional repression whereas in gene bodies, DNA methylation is generally enriched in the body of highly transcribed genes [61–64]. We tested the impacts of genome assembly quality on read mapping and DNA methylation calling, revealing DNA methylation peaks and valleys did change their locations and magnitudes, especially when they are near chromosome ends and sudden drops.

### PMDs

In this study, we reported large-scale PMDs in multiple cattle tissues. We then cross-referenced them on the chromosome level with CpG, genes, transcriptions, HMRs, histone codes, satellites, and TADs. We found that cattle PMDs share features with those identified in other species, especially those identified in human tissues: localization in genomic regions with low GC contents, low CGI density, low gene density, and lack of active histone marks. Although PMDs have been associated with gene repression and inactive chromatin marks, genes within tissue-specific PMDs did display tissue-specific functions. Previous human results show that PMDs are established within preformed TAD B compartments after cell lineage decision in cardiac myocytes [65]. The higher order chromatin conformation is proposed to be a regulatory mechanism guiding cell type-specific establishment of CpG methylation and non-CpG methylation signatures, like PMDs in TAD B compartments and HMR in TAD A compartments, respectively. Similarly, the endogenous bovine Hi-C contact maps uncovered that TAD B compartments were often associated with PMDs in the cattle genome. Thus, we hypothesize that a similar silencing mechanism may operate in cattle PMDs during cattle tissue specification and development.

### HMRs

We detected large differences between cattle somatic tissues in terms of HMRs. For example, the peak size of non-TSS-HMR for the placenta was significantly larger than those in sperm and normal tissues, while the peak sizes of TSS-HMR were highly consistent, (~ 2000 bp) among all tissue (Fig. 3a). This might indicate the dramatic difference of the placenta as compared to the sperm and other somatic tissues and the importance of the TSS-HMR throughout all tissues. We also classified genes into 5 groups according to their promoter location relative to the TSS-HMR and studied their potential impacts on gene regulation and genome evolution. By performing correlation analyses between the paired genes’ expressions, we found the paired genes (twin-genes) within TSS-HMR T2 would have more chances to be co-expressed (Fig. 4d). Moreover, our results showed that those paired genes were more consistent across mammalian species. As for genes within the TSS-HMR T3, i.e., with variable TSS or promoters, we found that they had a high possibility of being regulated by tissue-specific methylation of TSS-HMR. We used WGCNA to study gene networks based on pairwise correlations between their expressions and identified tissue-specific genes related to tissue functions. Combining with the gene expression using the RNA-seq data, we found 32 genes’ tissue-specific HMRs were highly correlated with their expression. The tissue-specific TSS-HMRs were greatly enriched for putative binding sites of transcription factors, which are known to have tissue-specific function (Fig. 4i). Combined with gene expression using the RNA-seq data, we identified tissue-specific gene expression correlated with tissue-specific HMR. Additionally, using our WGBS data, we totally identified 50,023 eCGIs at a single-base resolution and validated 42.24% of the total cCGI.

### Common repeats

In germ cells like sperm, common repeats are normally highly methylated. The conserved piRNA pathway has been proposed to be important for recognizing and silencing repeats in germ cells [66]. However, we still found more than expected HMRs that overlapped common repeats, suggesting some individual elements can evade piRNA-based silencing. Examining patterns of HMR-associated repeats is very informative. One possibility is that just like genes, young repeats contain promoters or regulatory regions and/or their TF binding and transcription activation can facilitate their evading default methylation. Although most of Bov-A2 elements follow the normal expectation, showing a negative correlation between methylation level and age (represented by their divergence from its consensus sequence), we detected that some Bov-A2 elements were hypomethylated in cattle sperm cells. Similar to the young Alu subfamilies, which introduce binding sites for transcription factor SABP in human sperm [67, 68], we found some Bov-A2 elements inserted into genes like *NME8* and *PHX4* that function in spermatogenesis or transcription regulation. Through examining these Bov-A2 insertions, we found the binding sites for multiple AZF1 (azoospermia factor 1), which have an essential meiotic function in fly and human spermatogenesis [69]. Diseases associated with AZF1 include azoospermia and varicocele [70]. As the introduction of TFBS by active Bov-A2 insertions could change the promoter structure, we hypothesize that Bov-A2 insertions in sperm cells may be involved in specific regulation of functional genes.

### Future directions and limitations

Genome editing technologies, CRISPR/Cas9, can directly target and edit individual methylation sites and therefore determine the exact function of DNA methylation at a specific site, as reviewed recently [71]. It is noted that because our data were produced from bulk cells, we were unable to determine the impact of cell composition on our results. Based on 64 human reference cell types, the human GTEx Consortium recently used the xCell method [72] to characterize the effect of cell type heterogeneity on analyses from bulk tissue [55]. Estimated cell type abundances from bulk RNA-seq across tissues reveal the cellular specificity of genetic regulation of gene expression across human tissues [73]. Due to limited resources, such as cattle reference cell types, future studies will be warranted to test these hypotheses and estimate their effects.

### Conclusions

In summary, using conventional WGBS and RNA-seq, we provided baseline methylation and transcription profiles for cattle somatic cells at a single-base resolution. We characterized the DNA methylome and assessed DNA methylation patterns. We reported rich data sets of PMDs and HMRs across different tissues and detected that some of them were correlated with tissue development. Our study contributes to the understanding of cattle DNA methylation patterns and provides foundational information for further investigations.

## Methods

### Sample collection, DNA and total RNA isolation, and sequencing

In this study, we collected 16 tissue types under the approval of the US Department of Agriculture, Agricultural Research Service, Beltsville Agricultural Research Center’s Institutional Animal Care and Use Committee (Protocol 16-016). Tissues were collected, snap frozen in liquid N_2_ immediately after excision, and kept at − 80 °C until use. Ten published samples were described before [24], including parenchymal tissue from the mammary glands, whole blood cells, and prefrontal cortex of the brain collected from two healthy adult Holstein cows (3–4 years old; one lactating and one non-lactating). Semen straws were collected twice from two fertile Holstein bulls. Among the newly generated data, we collected additional samples from the same two Holstein cows and their similar relatives based on a similar list as described before (Harhay et al., 2010). Genomic DNA for lung tissue was isolated according to the QIAamp DNA Mini Kit protocol (QIAGEN, Valencia, CA, USA). The quality of DNA samples was evaluated using the 2100 Bioanalyzer (Agilent Technologies, Santa Clara, CA, USA) including degradation, potential RNA contamination, purity (OD260/OD280), and concentration using a spectrophotometer (NanoDrop Technologies, Rockland, DE) to meet the requirements for library construction. We extracted the total RNA from snap-frozen tissues using TRIzol (Invitrogen, Carlsbad, CA, USA) according to the manufacturer’s instructions. We measured the quantity and purity of RNA using a NanoDrop 8000 Spectrophotometer (NanoDrop Technologies, Wilmington, DE) and Agilent 2100 Bioanalyzer System (Agilent Technologies). We contracted Novogene USA (Sacramento, CA, USA) to sequence these RNA samples using the Illumina HiSeq 2000 platform (Illumina, San Diego, CA) with paired-end (100 to150 bp) reads (Additional file 2: Table S1).

### WGBS library construction, sequencing, and identification of methylcytosine

The qualified genomic DNA from all samples were used to construct libraries. Briefly, 3 μg of genomic DNA spiked with unmethylated lambda DNA was fragmented into 200–300 bp using a Covaris S220 (Covaris, Inc., Woburn, MA, USA), followed by terminal repairing and A ligation. Different cytosine methylated barcodes were ligated to sonicated DNA for different samples. The DNA bisulfite conversion was performed using the EZ DNA Methylation Gold Kit (Zymo Research, Irvine, CA, USA). Then, single-stranded DNA fragments were amplified using the KAPA HiFi HotStart Uracil + ReadyMix (2 X) (Kapa Biosystems, Wilmington, MA, USA). The library concentration was quantified using a Qubit 2.0 fluorometer (Life Technologies, Carlsbad, CA, USA) and qPCR (iCycler, BioRad Laboratories, Hercules, CA, USA), and the insert size was checked using the Agilent 2100. To decrease the batch effect, the libraries for one sample were balanced, mixed with other libraries with different barcodes, and sequenced on different lanes of a HiSeq X Ten platform to generate 150-bp paired-end reads by Novogene (Novogene, Beijing, China).

Programs FastQC v 0.11.2 (FastQC) and Trim Galore v 0.4.0 (Trim Galore) were used to generate sequence quality reports and to trim/filter the sequences, respectively. For each sample, high-quality reads were obtained after trimming low-quality bases and the adapter sequences. The cleaned data for each sample were merged and aligned to the reference genome (ARS-UCD1.2) using bowtie2 under the Bismark software (0.14.5) with the parameters -p 3 -N 1 -D 20. The methylcytosine information was extracted using the bismark_methylation_extractor after deduplicating the duplication reads. The first 6 bp were ignored for the paired-end reads to decrease the potential effects of severe bias toward nonmethylation in the end-of-reads caused by end repairing.

### Genome sequencing library construction, sequencing, and identification of SNP

The lung DNA samples of the two Holstein cows were sequenced using the Illumina NextSeq550 platform, with the Nextera library preparation and sequence generation according to the manufacturer’s protocols. NGSQCToolkit v2.3.3 was used to trimmed adapter sequences and low-quality reads. All the clean reads were mapped on the reference genome (ARS-UCD1.2) using BWA v0.7.12 software. We only used the reliable mapped reads for SNP calling. The SNP positions within the aligned reads compared to the reference genome were detected using the pileup function in SAMtools v.1.7 utilities. SNPs were predicted with a minimum mapping quality (−Q) of 20 and with the minimum and maximum read depths of 3 and 100, respectively.

### RNA sequencing read alignment and assembly

The total RNA was first treated with DNase I to remove residual DNA. Then, poly(A) mRNA was isolated using beads with oligo(dT). The purified mRNA was first fragmented using the RNA fragmentation kit. First-strand cDNA synthesis was performed using random hexamer primers and reverse transcriptase. After the first strand was synthesized, a custom second-strand primer and strand synthesis buffer (Illumina) were both added, followed by dNTPs, RNase H, and DNA polymerase I to start the second-strand synthesis. Second, after a series of terminal repair, A ligation, and sequencing adaptor ligation, the double-stranded cDNA library is completed through size selection and PCR enrichment. Then, the cDNA libraries were prepared according to Illumina’s protocols and sequenced on the Illumina platform in Novogene USA, Sacramento, CA.

NGSQCToolkit v2.3.3 was used to trimmed adapter sequences and low-quality reads. The clean reads were aligned on the reference genome (ARS-UCD1.2) [51] along with annotated genes in the NCBI (ftp://ftp.ncbi.nlm.nih.gov/genomes/all/GCF/002/263/795/GCF_002263795.1_ARS-UCD1.2) using the HISAT2 v2.1.0 with the default parameters. The spliced reads were initially assembled to transcripts using the StringTie v1.3.3 software for each sample. Transcripts from all samples were merged to create a consensus reference transcriptome. The transcript per million mapped reads (TPM) and raw counts that mapped to the corresponding transcripts were estimated using the StringTie program.

### CpG island identification and validation

We used a distance-based algorithm to identify single-base resolution for CpG island on the cattle genome, following the CpGcluster software as described [17]. We calculated the methylation level of each computer-predicted CpG island (cCGI) for each sample. Only the CpG island with at least 5 CG detected with more than 5 × coverage for each sample was used for further experimental validation. The eCGI was defined as methylation level less than 30% in at least one sample.

### Histone mortification localization analysis for the cattle liver

ChIP-seq data for the cattle liver was downloaded from the Gene Expression Omnibus database with the accession number PRJEB6906. The sample preparation procedure can be found in [74]. NGSQCToolkit (version 2.3.3) software was used to filter the adapters and low-quality reads. Then, the qualified reads were aligned to the reference genome (ARS-UCD1.2) using bowtie2 (version 2.3.3; -N 0 -L 22 -i S,1,1.15 –dpad 15 -gbar 4), and peaks were called using MACS (version 1.4.2; –keep-dup 1 –wig –single-profile –space = 10 –diag) with default parameters.

### Identification of TAD using Hi-C data

The Hi-C data was retrieved from NCBI Sequence Read Archive under the accessions: SRR5753600, SRR5753603, and SRR5753606. These Hi-C libraries were prepared from the sequenced Hereford cow (Dominette) lung tissue and sequenced on an Illumina HiSeq 4000. NGSQCToolkit v2.3.3 was used to trimmed adapter sequences and low-quality reads. The clean reads were mapped on the reference genome (ARS-UCD1.2) using BWA software with parameters of mem -A1 -B4 -E50 -L0 -t 16. Hi-C matrices were imported to HiCExplorer v3.4.1 with the applications hicFindTADs and hicPlotTADs. Interaction frequency matrices at 50-kb resolution were transformed into *z*-score matrices based on the distribution of contacts at given genomic distances. The false discovery rate (FDR) was used to correct *P* values with threshold of 0.01. hicPlotTADs was used to plot specific regions for the interaction frequency matrices in combination with TAD boundary start and stop positions.

### PMD and HMR identification

We utilized the methpipe software (http://smithlabresearch.org/downloads/methpipe-manual.pdf) to identify PMD by applying an HMM model to each sample. To detect the large PMDs accurately, we used different window sizes (5 kb, 10 kb, 20 kb, 50 kb) to generate the PMD localizations and examined the result by randomly selected visualization as recommended by the manual of the methpipe software. Finally, we chose 10-kb window size for all the samples. The HMR was identified following the manual of the methpipe software with the default parameters.

### Global comparison between methylomes of different samples

The common CGs with depth greater than 10 × among all samples were used for global comparison between each of the two sample pairs. Detections of DMC and DMR were applied using an R package (methykit, R version 3.3.3). The DMCs were defined as the methylation difference greater than 30% and *q* value < 0.05. The DMRs were defined as the average methylation difference greater than 30% and *q* value < 0.05 using a 500-bp window size. To receive more accurate DMRs, we only selected the DMRs that supported by at least 5 DMCs in the same direction for analyses (Additional file 2: Table S3 Column D). To call the tissue-specific DMRs, we ranked all DMRs by their frequencies derived from all pairwise comparisons. We then chose the top 0.01~0.3% of the DMRs (by considering the GO enrichment results) and merge them into the final nonredundant tissue-specific DMRs, which showed significant differences between the tissue to all other samples.

The genome structure annotation files for genes and repeats were downloaded from the NCBI database (ftp://ftp.ncbi.nlm.nih.gov/genomes/all/GCF/002/263/795/GCF_002263795.1_ARS-UCD1.2) [51]. The promoter regions were defined as ± 1000 bp around the transcript start sites. The methylation levels for each element in different genomic features were calculated as the average methylation level of the CGs with at least 5 × coverage. Only the elements that met the following criteria were used for further analysis: at least 10% CG detection rate for elements with more than 50 CGs and at least five CGs detected for elements with fewer than 50 CGs. R packages were used to plot the comparison results.

### Gene function analysis

Gene functional annotation analyses were applied using the online DAVID software. The Fisher exact test was used to measure gene enrichment in annotation terms. *P* values were corrected by FDR to search for significantly enriched terms. We used Homer software to detect enriched motifs within the tissue-specific TSS-HMR of genes. The MEME online software (http://meme-suite.org/) [75] was used to enrich the significant different motif between the neCGI and eCGI.

## Supplementary information

**Additional file 1: Figure S1.** Comparison of methylation distribution between two different cattle genome reference assemblies using a sperm sample as an example. Blue line: UMD3.1.1; Green line: ARS-UCD1.2, from top left to down right: chr1-chr29. **Figure S2.** Comparison of methylation distribution between two different cattle genome reference assemblies using chr28 as an example for all samples. Blue line: UMD3.1.1; Green line: ARS-UCD1.2. **Figure S3.** Principal component analysis (PCA) for all samples using DNA methylation levels of 500 bp windows: a, PC1 vs. PC2; b, PC1 vs. PC3; and c, PC1 vs. PC4. **Figure S4.** Correlation analyses for all samples using DNA methylation. **Figure S5.** Correlation analyses of DNA methylation using different window size. 20 K, 100 K, 500 K and 1 M refer to window sizes of 20 kb, 100 kb, 500 kb and 1 Mb, respectively. **Figure S6.** Standard division of methylation level across all samples. We defined methylation conserved and variable regions as the bottom and top tails, respectively. **Figure S7.** Genome features enrichment in hypermethylation conserved regions, including genic region and neCpG island. **Figure S8.** Gene ontology analyses for the genes located in the methylation level conserved and variable regions. (a) Genes located in the hypermethylation conserved regions; (b) Genes located in the sperm hypomethylation variable regions; (c) Genes located in the sperm hypermethylation variable regions. **Figure S9.** Genome features enrichment in hypomethylation conserved regions, including promoters, eCpG islands, and tRNA genes. **Figure S10.** Genome features enrichment in methylation variable regions, including promoter and eCpG island. Note: the enrichment of the cCpG island is mainly caused by eCpG island. **Figure S11.** GO analysis for the genes overlapped with methylation variable regions. **Figure S12.** Heatmap analysis using the methylation variable regions. **Figure S13.** Comparison of gene expressions between genes located in the PMD and non-PMD. **Figure S14.** Placenta HMDs, as compared to placenta PMDs, were enriched for the gene-related features including genic regions, promoters, eCGI and RefGenes. **Figure S15.** Comparison of methylation patterns for the gene and the CGI regions between HMD and PMD. Placenta HMDs showed significantly lower methylation patterns around the gene TSS and the CGI, while placenta PMDs were almost indistinguishable from the flanking backgrounds because of their low methylations. **Figure S16.** Comparison of HMR in terms of location and size among the different clusters. **Figure S17.** Genome feature enrichment in the HMRs of different clusters. **Figure S18.** Liver (a) and kidney (b) genes with conserved methylated TSS-HMR between cattle and human were involved in basic biological processes, like RNA processing, protein folding and cell cycle. **Figure S19.** Comparison of the TSS-HMR core region and the TSS-HMR two flank regions in terms of (a) the CG density; (b) standard deviations of the methylation level; (c) DMC distribution; and (d) methylation level. **Figure S20.** WGCNA analysis for the RNA sequencing data. (a) cluster dendrogram of the gene expression; (b) heatmap plot for the expression of tissue specific high expression genes. **Figure S21.** Genome distribution of the eCpG island. (a) Distribution of the eCpG island on the 29 cattle chromosomes. (b) Genomic features enrichment in eCpG island, including promoter (1000 bp around the TSS) and the first exon. **Figure S22.** The methylation pattern between the eCGI and the neCGI. (a) heatmap of the cCGI methylation level for all samples; the blue bar: eCGI; the green bar: neCGI; (b) comparison of the methylation patterns between the eCGI and the neCGI. **Figure S23.** Correlation analysis for eCGIs with (a) gene contents and (b) chromosome lengths. **Figure S24.** Comparison of the distribution on chromosomes between eCGI and the neCGI; red line: eCGI; green line: neCGI. **Figure S25.** CT heterozygote rate for the two animals in different CGIs; CT heterozygote represent CT and GA heterozygote when consider the two strands of DNA. **Figure S26.** Methylation level distributions for different genome features and samples. **Figure S27.** Distributions of 4389 common repeats located in the TSS-HMR.

**Additional file 2: Table S1A.** Sample information for the WGBS; **Table S1B.** Sample information for the RNA sequencing; **Table S1C.** Sample information for the whole genome sequencing; **Table S2.** Comparison of methylation statistics between two different cattle reference assemblies. **Table S3.** DMC and DMR number for each comparison pairs. Please note that the numbers, which may be affected by different common data amount for each tissue. **Table S4.** PMD information for different samples. **Table S5.** Placenta PMD percentages and genes located in the shared and lineage-specific placenta PMDs between cattle and human. **Table S6.** GO analyses for genes shared or specific in PMDs between human placenta and cattle placenta. **Table S7.** Gene ontology analysis for genes overlapping with cattle placenta HMD. **Table S8.** GO analysis for the genes overlapped with cattle placenta PMDs. **Table S9.** Information of 16 non-blood DNA methylation level drops. **Table S10.** Differentially methylated TSS-HMR between human and cattle. **Table S11.** Gene ontology analysis for genes totally located in the HMR. **Table S12**. common twin genes in one HMR core region between cattle and human. **Table S13.** Gene ontology analyses for genes specifically high expressed in different tissues. **Table S14.** Information for experimentally supported CpG Islands.

## Data Availability

All cattle RNA-seq (GSE137943) [76], WGBS (GSE147087) [77], and WGS data (GSE146345) [78] generated by this study are available from NCBI Gene Expression Omnibus (GEO; http://www.ncbi.nlm.nih.gov/geo/). Human WGBS datasets of the liver (SRR3269859), kidney, (SRR1654399, SRR1654400, and SRR1764401) [74], and placenta (SRX381710) [13] were downloaded from NCBI Sequence Read Archive, respectively.

## Abbreviations

CGI: CpG island
DMC: Differentially methylated cytosine
FAANG: Functional Annotation of Animal Genome project
FDR: False discovery rate
GO: Gene Ontology
HMR: Hypomethylated region
LINE: Long interspersed nuclear element
LTR: Long terminal repeat
PMD: Partially methylated domain
O/E: Observed/expected
RRBS: Reduced representation bisulfite sequencing
SINE: Short interspersed nuclear element
TFBS: Transcription factor binding site
TSS: Transcription start site
WGBS: Whole genome bisulfite sequencing
