# Supplementary material for: Comparative whole genome DNA methylation profiling across cattle tissues reveals global and tissue-specific methylation patterns
Source: BMC Biol. 2020 Jul 6;18:85. doi: 10.1186/s12915-020-00793-5 (PMC7339546; doi:10.1186/s12915-020-00793-5)
Supplement: Supplementary file 1 — Additional file 1: Figure S1. Comparison of methylation distribution between two different cattle genome reference assemblies using a sperm sample as an example. Blue line: UMD3.1.1; Green line: ARS-UCD1.2, from top left to down right: chr1-chr29. Figure S2. Comparison of methylation distribution between two different cattle genome reference assemblies using chr28 as an example for all samples. Blue line: UMD3.1.1; Green line: ARS-UCD1.2. Figure S3. Principal component analysis (PCA) for all samples using DNA methylation levels of 500 bp windows: a, PC1 vs. PC2; b, PC1 vs. PC3; and c, PC1 vs. PC4. Figure S4. Correlation analyses for all samples using DNA methylation. Figure S5. Correlation analyses of DNA methylation using different window size. 20 K, 100 K, 500 K and 1 M refer to window sizes of 20 kb, 100 kb, 500 kb and 1 Mb, respectively. Figure S6. Standard division of methylation level across all samples. We defined methylation conserved and variable regions as the bottom and top tails, respectively. Figure S7. Genome features enrichment in hypermethylation conserved regions, including genic region and neCpG island. Figure S8. Gene ontology analyses for the genes located in the methylation level conserved and variable regions. (a) Genes located in the hypermethylation conserved regions; (b) Genes located in the sperm hypomethylation variable regions; (c) Genes located in the sperm hypermethylation variable regions. Figure S9. Genome features enrichment in hypomethylation conserved regions, including promoters, eCpG islands, and tRNA genes. Figure S10. Genome features enrichment in methylation variable regions, including promoter and eCpG island. Note: the enrichment of the cCpG island is mainly caused by eCpG island. Figure S11. GO analysis for the genes overlapped with methylation variable regions. Figure S12. Heatmap analysis using the methylation variable regions. Figure S13. Comparison of gene expressions between genes located in the PMD and non-PMD. Figure [file 12915_2020_793_MOESM1_ESM.pdf]

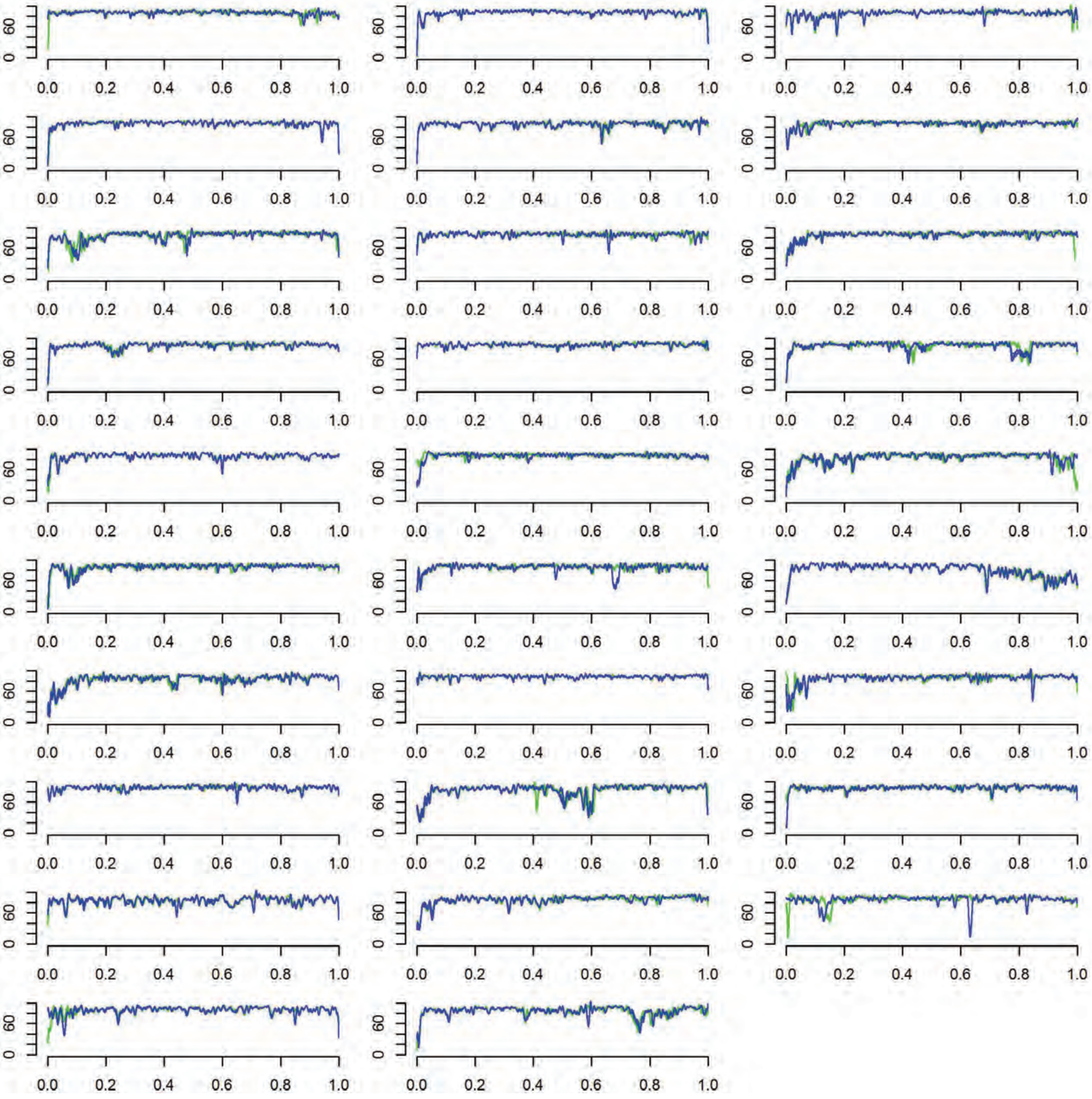


Figure S1

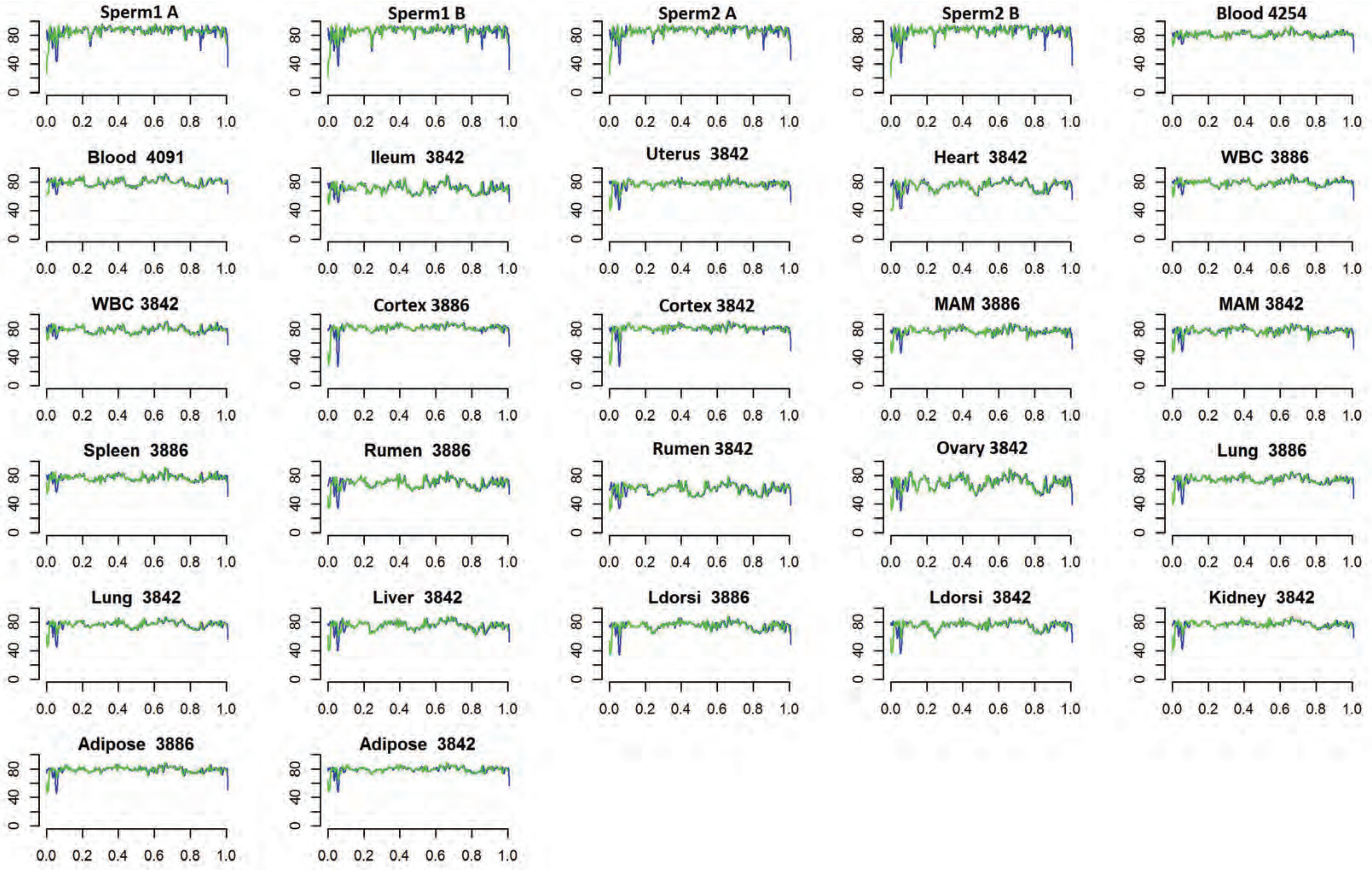
Sperm1 A
Sperm1 B
Sperm2 A
Sperm2 B
Blood 4254
Blood 4091
Ileum 3842
Uterus 3842
Heart 3842
WBC 3886
WBC 3842
Cortex 3886
Cortex 3842
MAM 3886
MAM 3842
Spleen 3886
Rumen 3886
Rumen 3842
Ovary 3842
Lung 3886
Lung 3842
Liver 3842
Ldorsi 3886
Ldorsi 3842
Kidney 3842
Adipose 3886
Adipose 3842
0
40
80
0.0
0.2
0.4
0.6
0.8
1.0

Figure S2

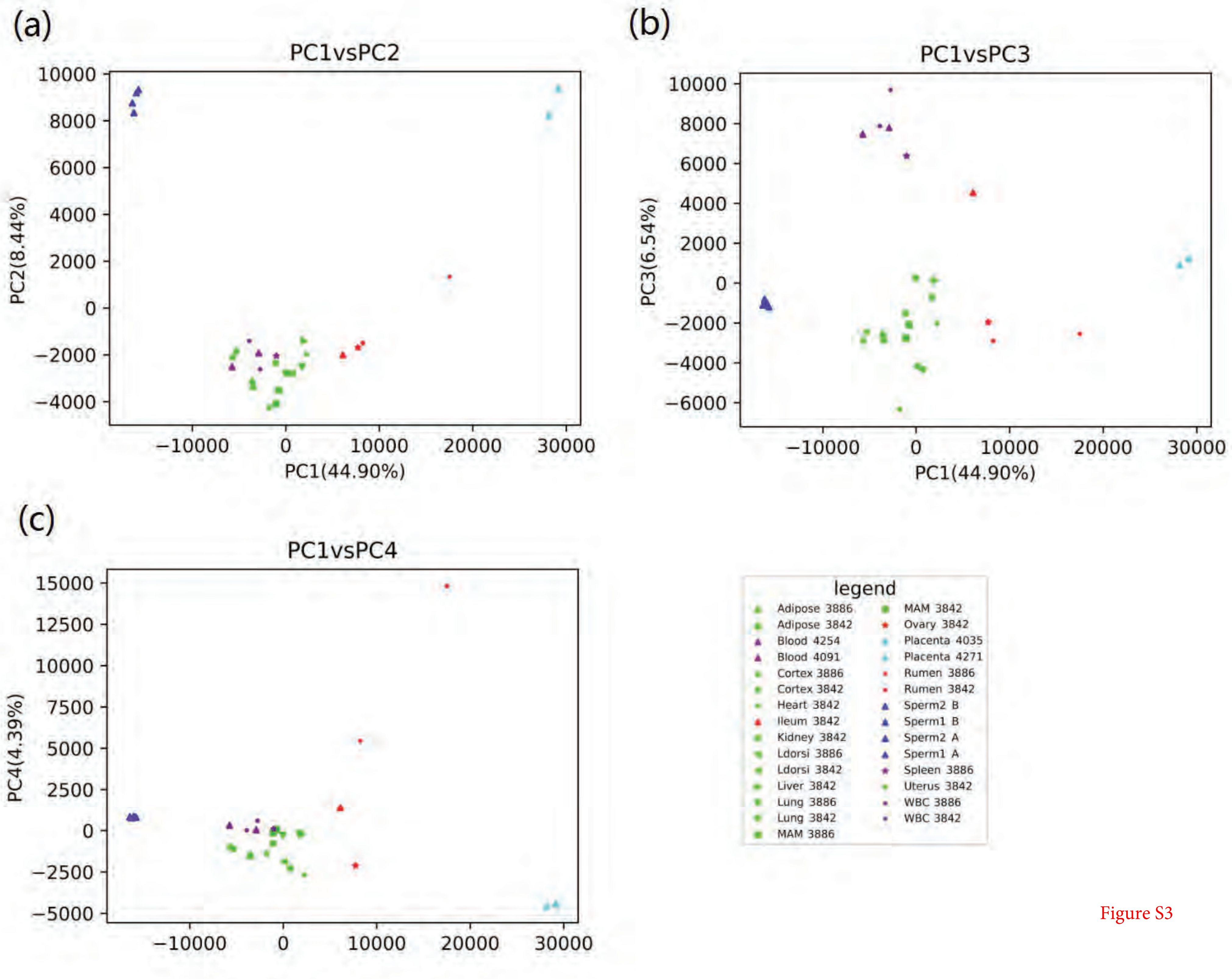

(a)
PC1vsPC2
PC2(8.44%)
PC1(44.90%)
(b)
PC1vsPC3
PC3(6.54%)
PC1(44.90%)
(c)
PC1vsPC4
PC4(4.39%)
legend
Adipose 3886
Adipose 3842
Blood 4254
Blood 4091
Cortex 3886
Cortex 3842
Heart 3842
Ileum 3842
Kidney 3842
Ldorsi 3886
Ldorsi 3842
Liver 3842
Lung 3886
Lung 3842
MAM 3886
MAM 3842
Ovary 3842
Placenta 4035
Placenta 4271
Rumen 3886
Rumen 3842
Sperm2 B
Sperm1 B
Sperm2 A
Sperm1 A
Spleen 3886
Uterus 3842
WBC 3886
WBC 3842


Figure S3

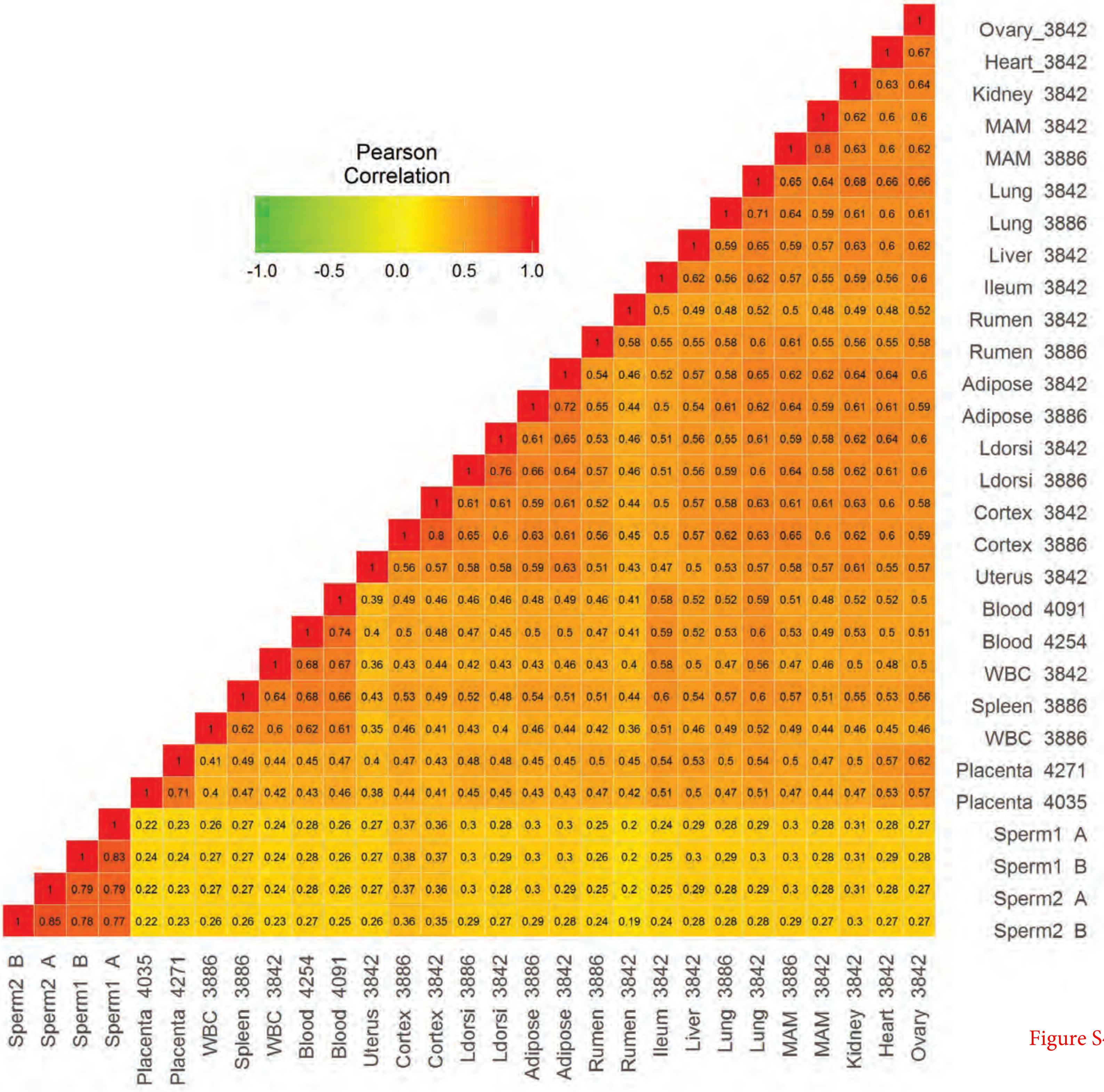
Pearson Correlation
-1.0
-0.5
0.0
0.5
1.0
Ovary_3842
Heart_3842
Kidney 3842
MAM 3842
MAM 3886
Lung 3842
Lung 3886
Liver 3842
Ileum 3842
Rumen 3842
Rumen 3886
Adipose 3842
Adipose 3886
Ldorsi 3842
Ldorsi 3886
Cortex 3842
Cortex 3886
Uterus 3842
Blood 4091
Blood 4254
WBC 3842
Spleen 3886
WBC 3886
Placenta 4271
Placenta 4035
Sperm1 A
Sperm1 B
Sperm2 A
Sperm2 B

Figure S4

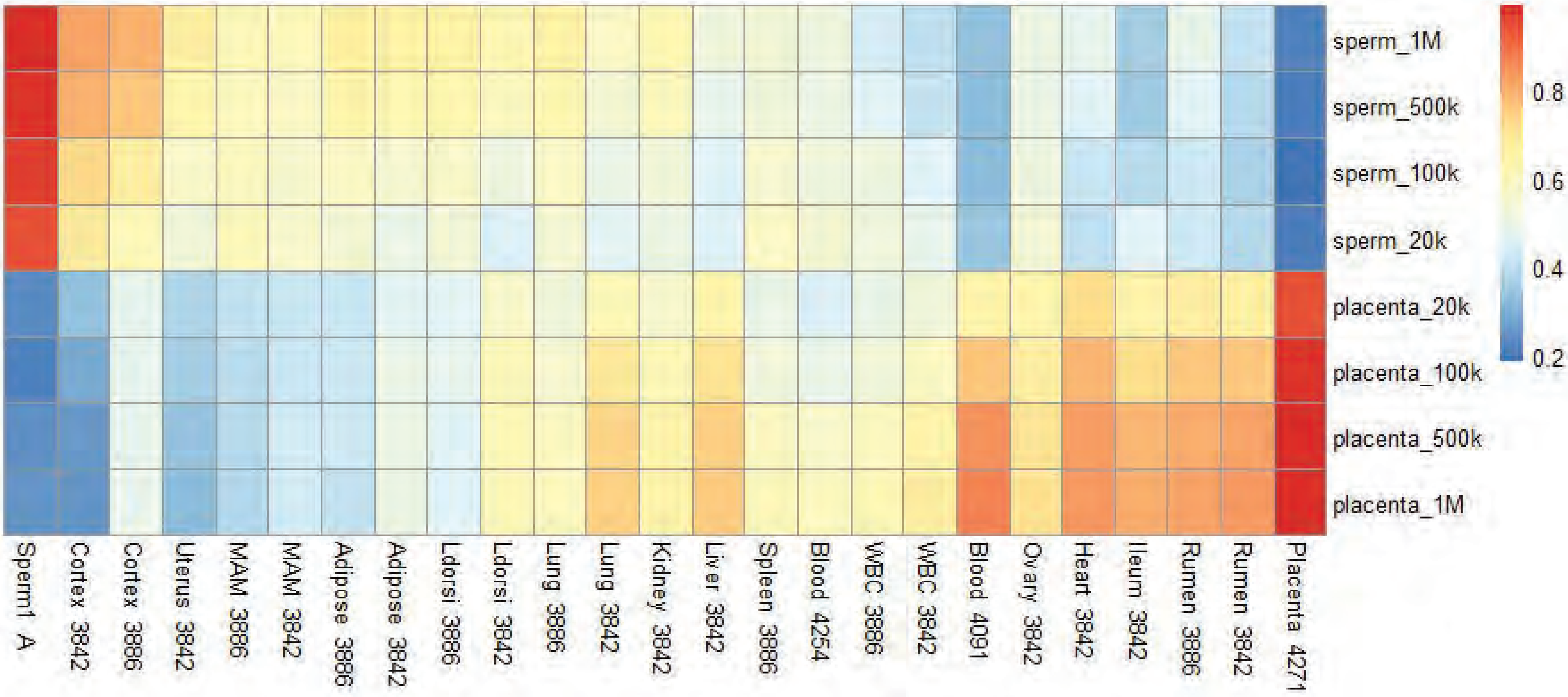

sperm_1M
sperm_500k
sperm_100k
sperm_20k
placenta_20k
placenta_100k
placenta_500k
placenta_1M
0.8
0.6
0.4
0.2
Sperm1 A
Cortex 3842
Cortex 3886
Uterus 3842
MAM 3886
MAM 3842
Adipose 3886
Adipose 3842
Ldorsi 3886
Ldorsi 3842
Lung 3886
Lung 3842
Kidney 3842
Liver 3842
Spleen 3886
Blood 4254
WBC 3886
WBC 3842
Blood 4091
Ovary 3842
Heart 3842
Ileum 3842
Rumen 3886
Rumen 3842
Placenta 4271


Figure S5

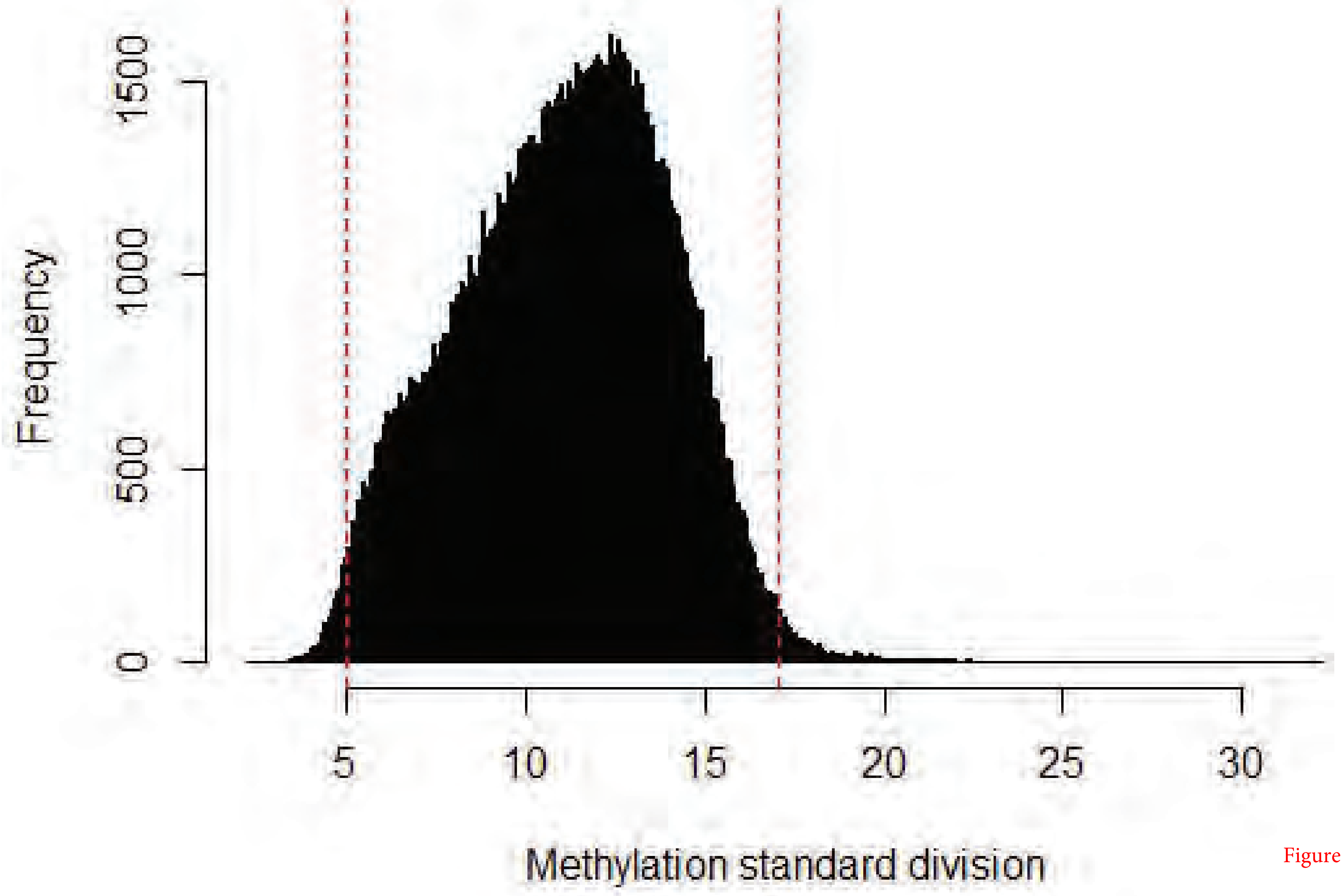

Frequency
0
500
1000
1500
5
10
15
20
25
30
Methylation standard division


Figure S6

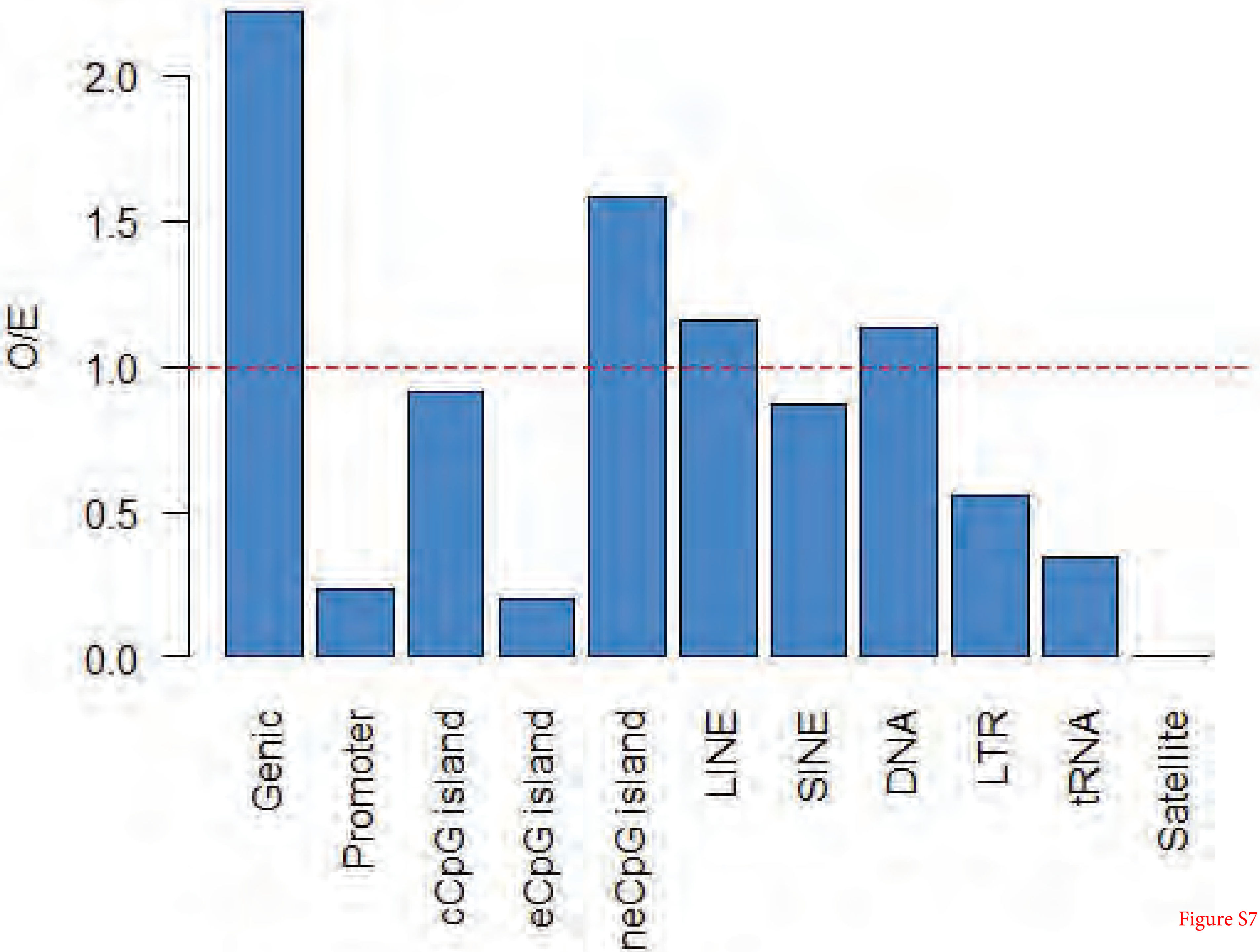

O/E
2.0
1.5
1.0
0.5
0.0
Genic
Promoter
cCpG island
eCpG island
neCpG island
LINE
SINE
DNA
LTR
tRNA
Satellite


Figure S7

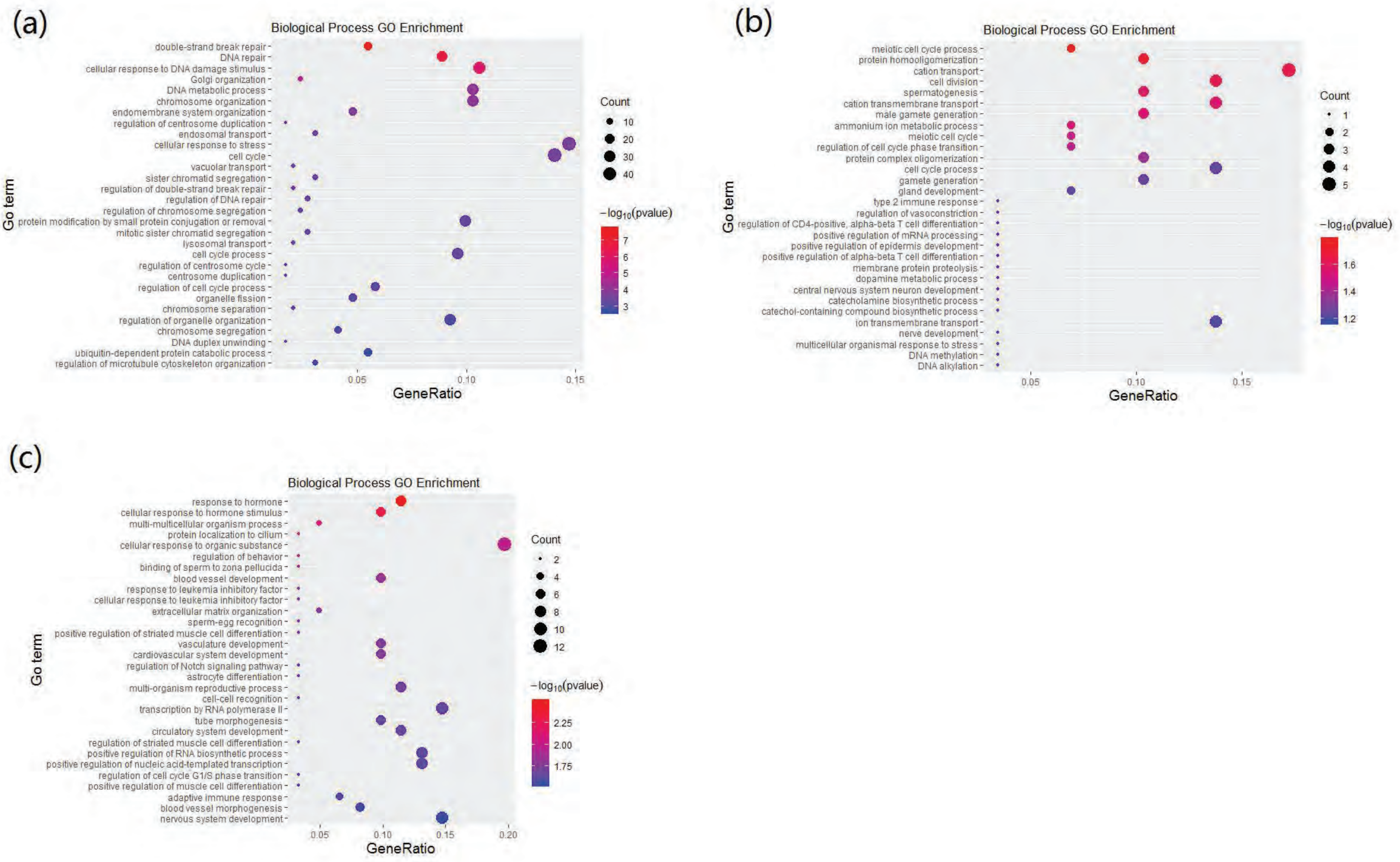
(a)
Biological Process GO Enrichment
double-strand break repair
DNA repair
cellular response to DNA damage stimulus
Golgi organization
DNA metabolic process
chromosome organization
endomembrane system organization
regulation of centrosome duplication
endosomal transport
cellular response to stress
cell cycle
vacuolar transport
sister chromatid segregation
regulation of double-strand break repair
regulation of DNA repair
regulation of chromosome segregation
protein modification by small protein conjugation or removal
mitotic sister chromatid segregation
lysosomal transport
cell cycle process
regulation of centrosome cycle
centrosome duplication
regulation of cell cycle process
organelle fission
chromosome separation
regulation of organelle organization
chromosome segregation
DNA duplex unwinding
ubiquitin-dependent protein catabolic process
regulation of microtubule cytoskeleton organization
Go term
0.05
0.10
0.15
GeneRatio
Count
10
20
30
40
$-\log_{10}(\text{pvalue})$
7
6
5
4
3
(b)
Biological Process GO Enrichment
meiotic cell cycle process
protein homooligomerization
cation transport
cell division
spermatogenesis
cation transmembrane transport
male gamete generation
ammonium ion metabolic process
meiotic cell cycle
regulation of cell cycle phase transition
protein complex oligomerization
cell cycle process
gamete generation
gland development
type 2 immune response
regulation of vasoconstriction
regulation of CD4-positive, alpha-beta T cell differentiation
positive regulation of mRNA processing
positive regulation of epidermis development
positive regulation of alpha-beta T cell differentiation
membrane protein proteolysis
dopamine metabolic process
central nervous system neuron development
catecholamine biosynthetic process
catechol-containing compound biosynthetic process
ion transmembrane transport
nerve development
multicellular organismal response to stress
DNA methylation
DNA alkylation
Go term
0.05
0.10
0.15
GeneRatio
Count
1
2
3
4
5
$-\log_{10}(\text{pvalue})$
1.6
1.4
1.2
(c)
Biological Process GO Enrichment
response to hormone
cellular response to hormone stimulus
multi-multicellular organism process
protein localization to cilium
cellular response to organic substance
regulation of behavior
binding of sperm to zona pellucida
blood vessel development
response to leukemia inhibitory factor
cellular response to leukemia inhibitory factor
extracellular matrix organization
sperm-egg recognition
positive regulation of striated muscle cell differentiation
vasculature development
cardiovascular system development
regulation of Notch signaling pathway
astrocyte differentiation
multi-organism reproductive process
cell-cell recognition
transcription by RNA polymerase II
tube morphogenesis
circulatory system development
regulation of striated muscle cell differentiation
positive regulation of RNA biosynthetic process
positive regulation of nucleic acid-templated transcription
regulation of cell cycle G1/S phase transition
positive regulation of muscle cell differentiation
adaptive immune response
blood vessel morphogenesis
nervous system development
Go term
0.05
0.10
0.15
0.20
GeneRatio
Count
2
4
6
8
10
12
$-\log_{10}(\text{pvalue})$
2.25
2.00
1.75

Figure S8

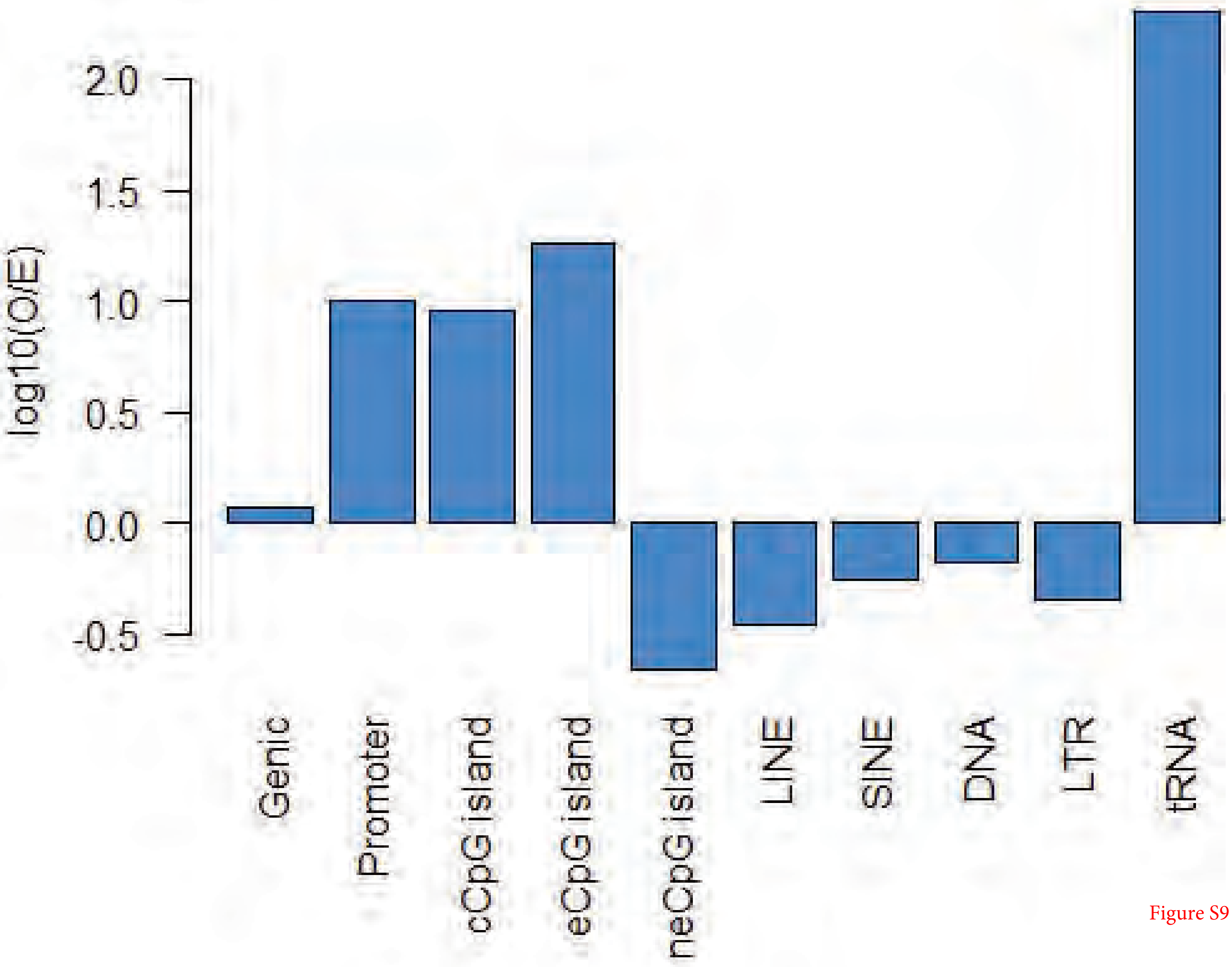
log10(O/E)
2.0
1.5
1.0
0.5
0.0
-0.5
Genic
Promoter
cCpG island
eCpG island
neCpG island
LINE
SINE
DNA
LTR
tRNA

Figure S9

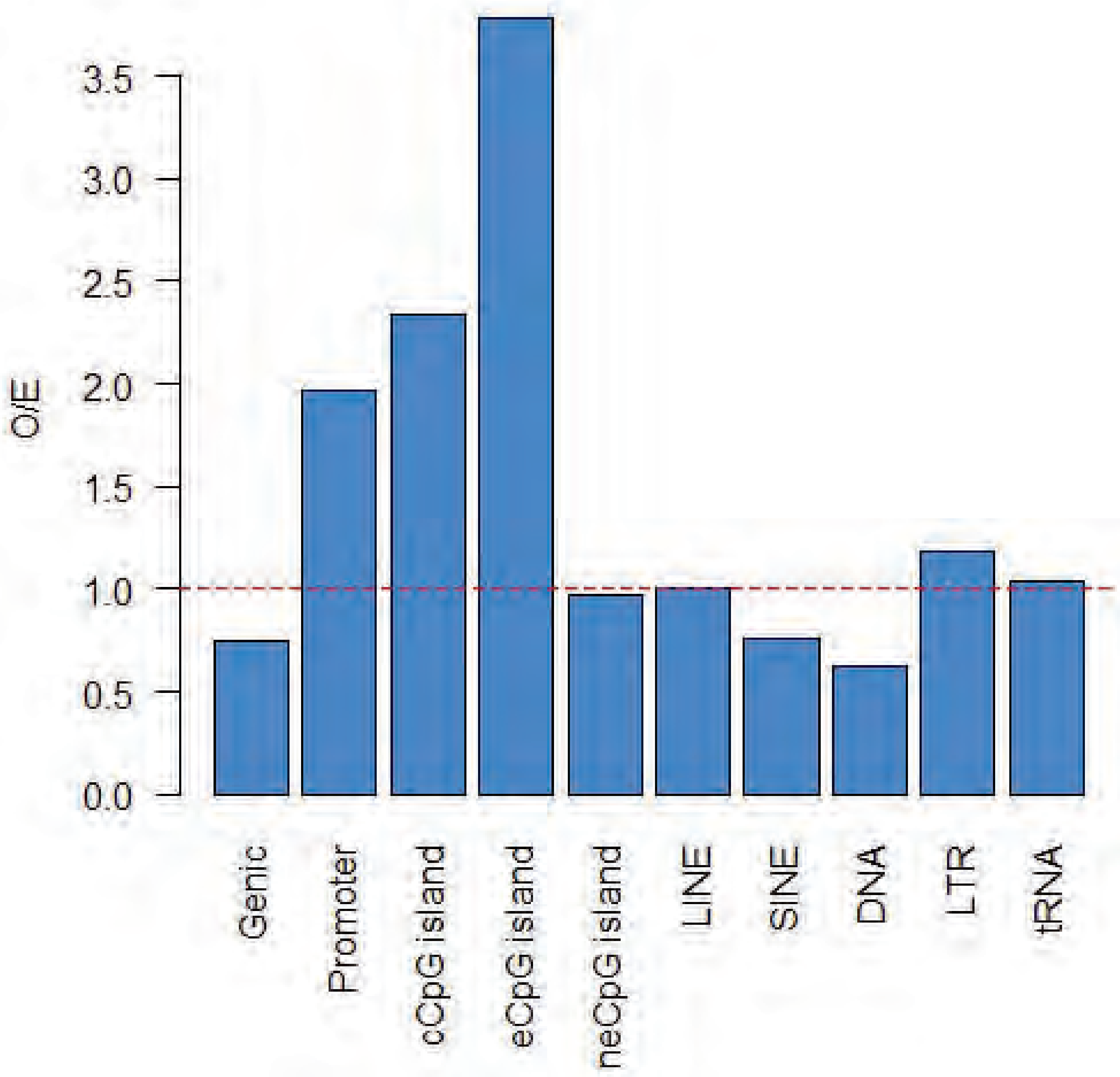
O/E
0.0
0.5
1.0
1.5
2.0
2.5
3.0
3.5
Genic
Promoter
cCpG island
eCpG island
neCpG island
LINE
SINE
DNA
LTR
tRNA

Figure S10

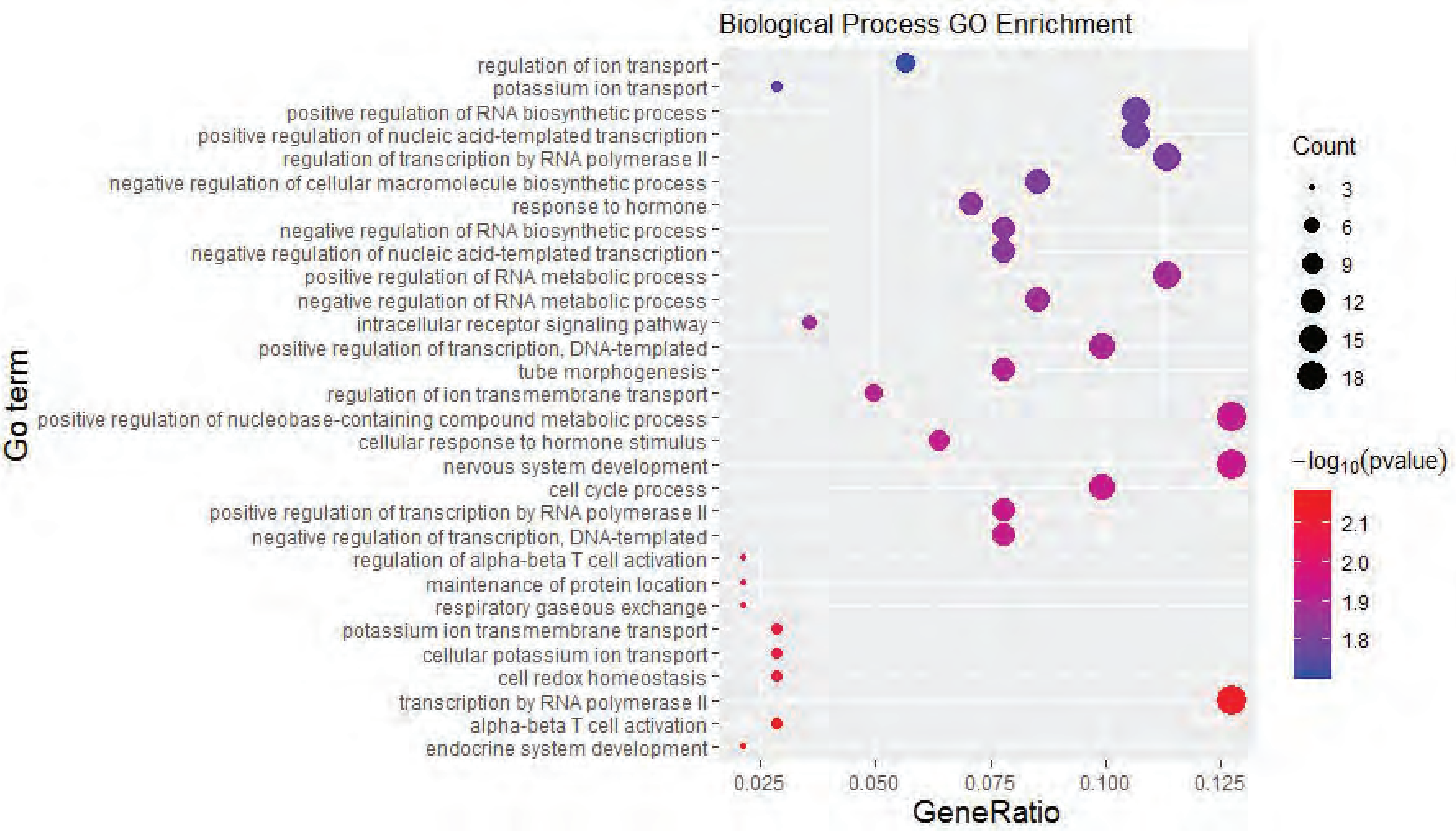
Biological Process GO Enrichment
Go term
regulation of ion transport
potassium ion transport
positive regulation of RNA biosynthetic process
positive regulation of nucleic acid-templated transcription
regulation of transcription by RNA polymerase II
negative regulation of cellular macromolecule biosynthetic process
response to hormone
negative regulation of RNA biosynthetic process
negative regulation of nucleic acid-templated transcription
positive regulation of RNA metabolic process
negative regulation of RNA metabolic process
intracellular receptor signaling pathway
positive regulation of transcription, DNA-templated
tube morphogenesis
regulation of ion transmembrane transport
positive regulation of nucleobase-containing compound metabolic process
cellular response to hormone stimulus
nervous system development
cell cycle process
positive regulation of transcription by RNA polymerase II
negative regulation of transcription, DNA-templated
regulation of alpha-beta T cell activation
maintenance of protein location
respiratory gaseous exchange
potassium ion transmembrane transport
cellular potassium ion transport
cell redox homeostasis
transcription by RNA polymerase II
alpha-beta T cell activation
endocrine system development
0.025
0.050
0.075
0.100
0.125
GeneRatio
Count
3
6
9
12
15
18
$-\log_{10}$(pvalue)
2.1
2.0
1.9
1.8

Figure S11

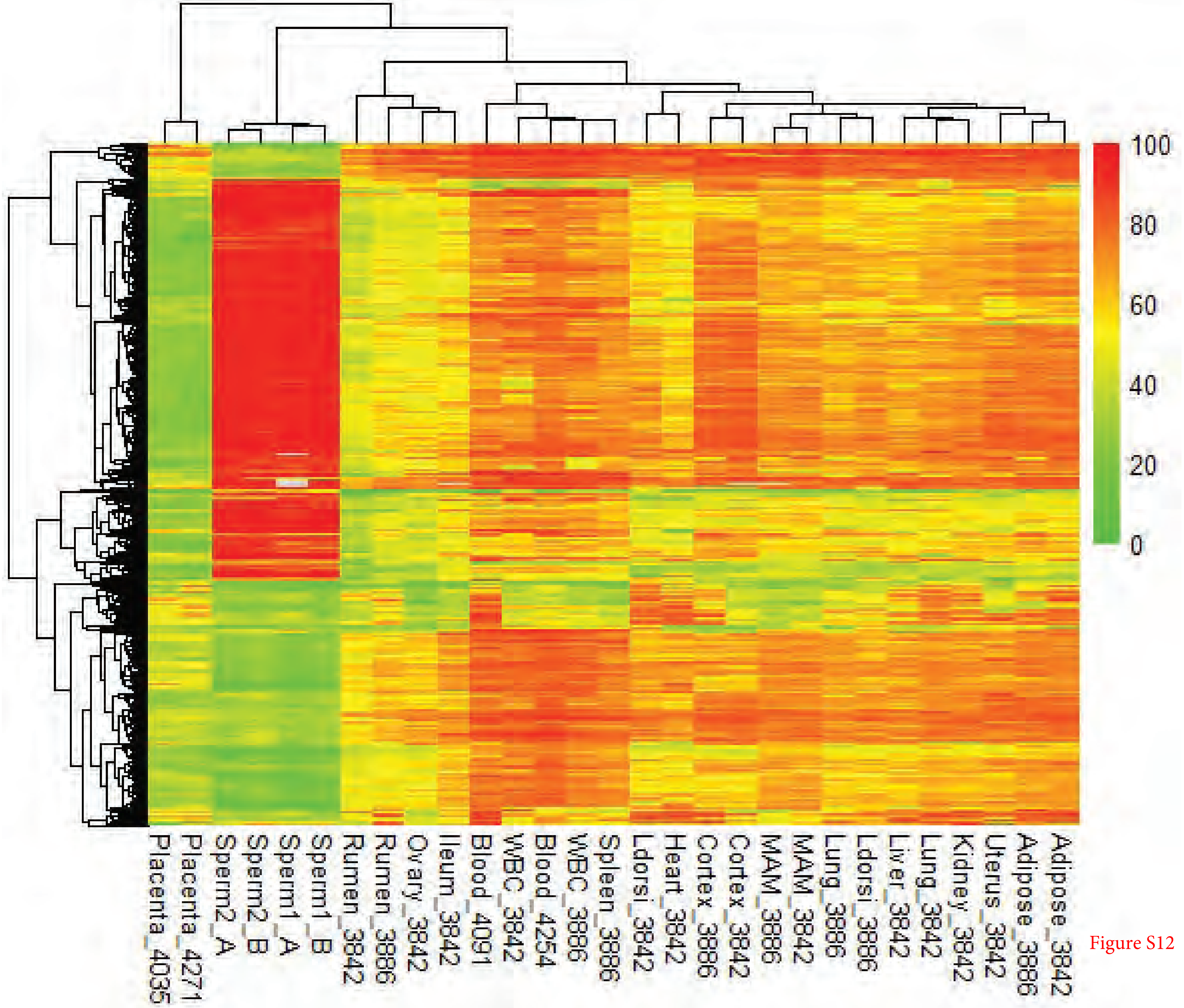
100
80
60
40
20
0
Placenta_4035
Placenta_4271
Sperm2_A
Sperm2_B
Sperm1_A
Sperm1_B
Rumen_3842
Rumen_3886
Ovary_3842
Ileum_3842
Blood_4091
WBC_3842
Blood_4254
WBC_3886
Spleen_3886
Ldorsi_3842
Heart_3842
Cortex_3886
Cortex_3842
MAM_3886
MAM_3842
Lung_3886
Ldorsi_3886
Liver_3842
Lung_3842
Kidney_3842
Uterus_3842
Adipose_3886
Adipose_3842

Figure S12

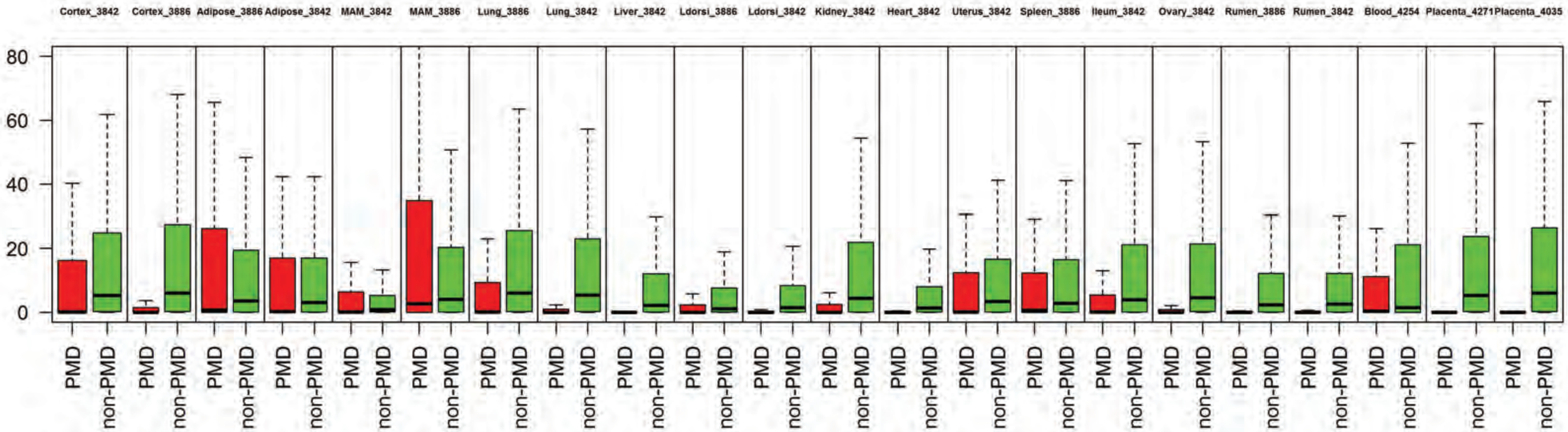
Cortex_3842
Cortex_3886
Adipose_3886
Adipose_3842
MAM_3842
MAM_3886
Lung_3886
Lung_3842
Liver_3842
Ldorsi_3886
Ldorsi_3842
Kidney_3842
Heart_3842
Uterus_3842
Spleen_3886
Ileum_3842
Ovary_3842
Rumen_3886
Rumen_3842
Blood_4254
Placenta_4271
Placenta_4035
0
20
40
60
80
PMD
non-PMD

Figure S13

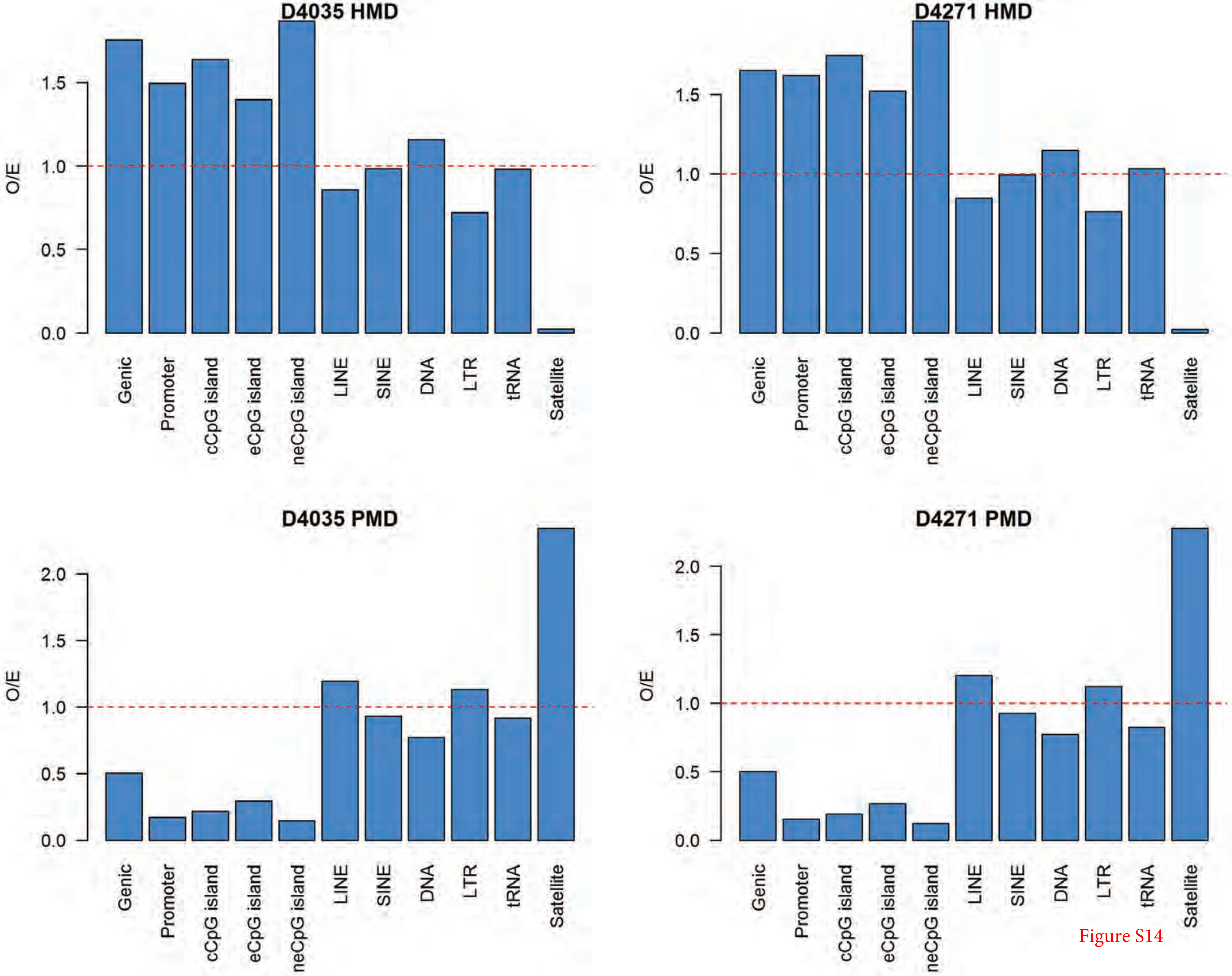

D4035 HMD
D4271 HMD
D4035 PMD
D4271 PMD
O/E
0.0
0.5
1.0
1.5
2.0
Genic
Promoter
cCpG island
eCpG island
neCpG island
LINE
SINE
DNA
LTR
tRNA
Satellite


Figure S14

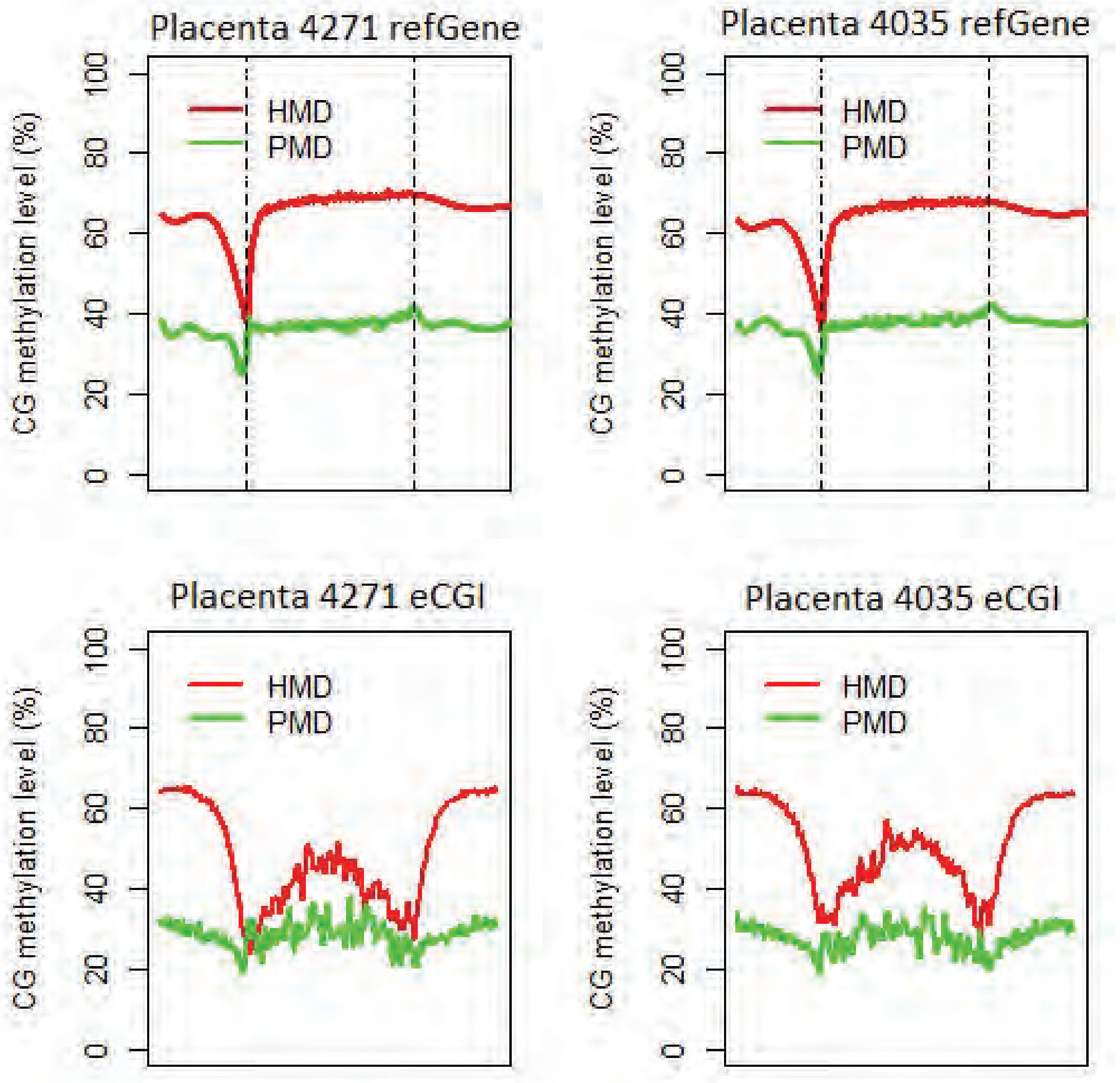

Placenta 4271 refGene
Placenta 4035 refGene
Placenta 4271 eCGI
Placenta 4035 eCGI
CG methylation level (%)
0
20
40
60
80
100
HMD
PMD


Figure S15

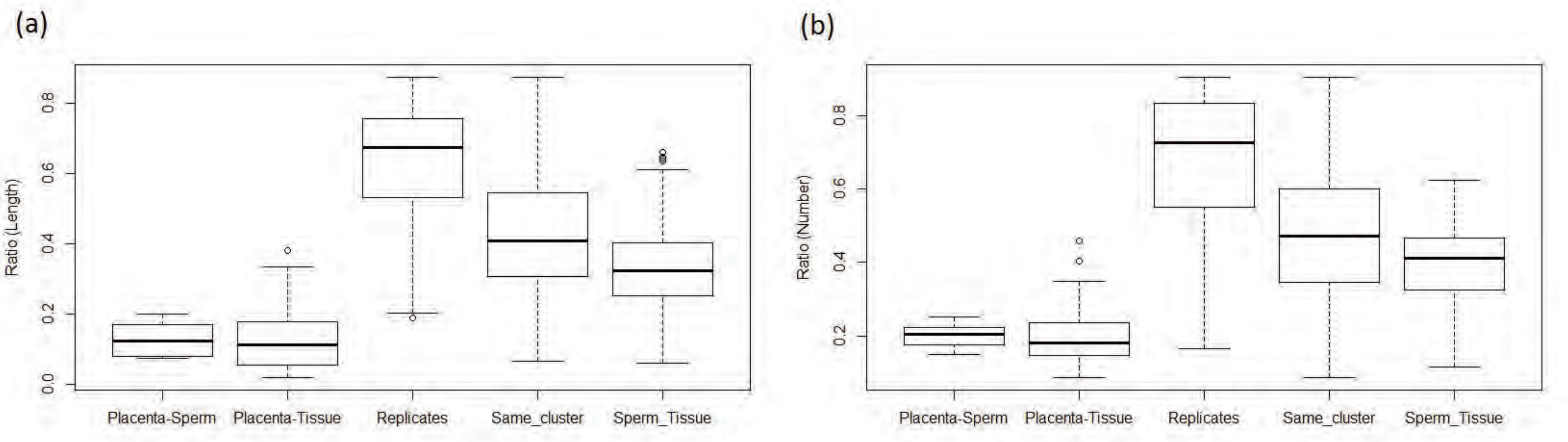
(a)
Ratio (Length)
0.0
0.2
0.4
0.6
0.8
Placenta-Sperm
Placenta-Tissue
Replicates
Same_cluster
Sperm_Tissue
(b)
Ratio (Number)
0.2
0.4
0.6
0.8
Placenta-Sperm
Placenta-Tissue
Replicates
Same_cluster
Sperm_Tissue

Figure S16

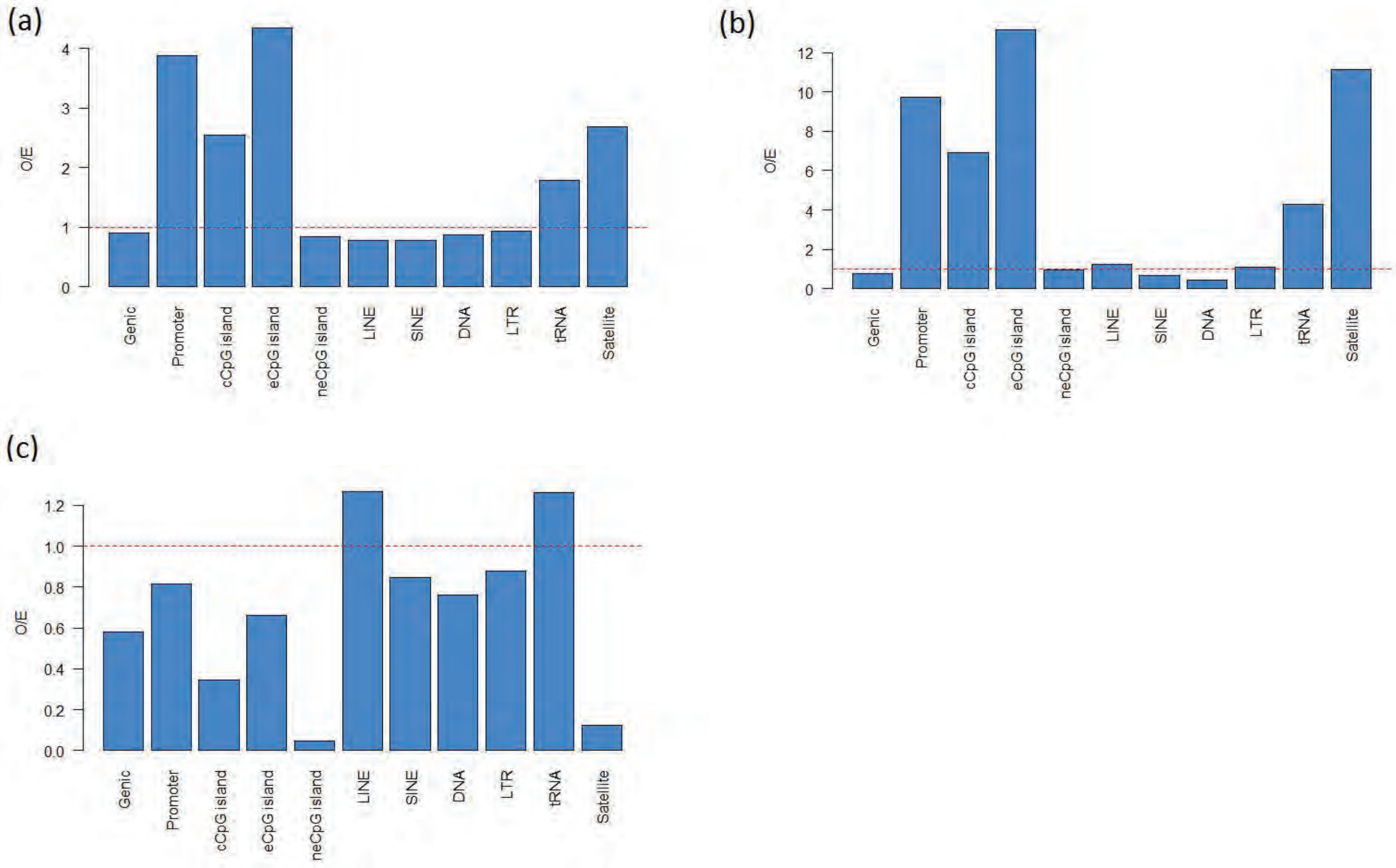

(a)
O/E
0
1
2
3
4
Genic
Promoter
cCpG island
eCpG island
neCpG island
LINE
SINE
DNA
LTR
tRNA
Satellite
(b)
O/E
0
2
4
6
8
10
12
Genic
Promoter
cCpG island
eCpG island
neCpG island
LINE
SINE
DNA
LTR
tRNA
Satellite
(c)
O/E
0.0
0.2
0.4
0.6
0.8
1.0
1.2
Genic
Promoter
cCpG island
eCpG island
neCpG island
LINE
SINE
DNA
LTR
tRNA
Satellite


Figure S17

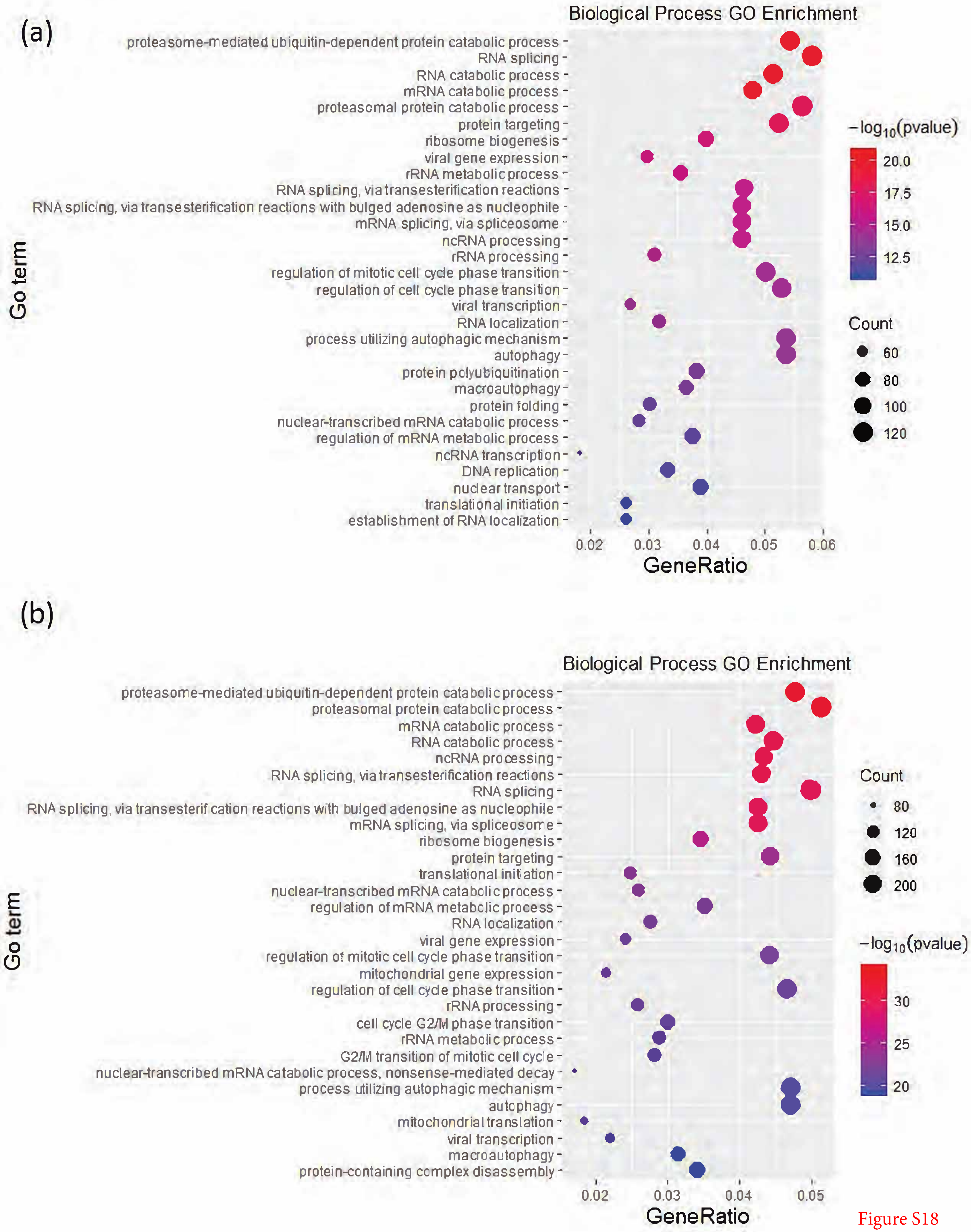
(a)
Biological Process GO Enrichment
proteasome-mediated ubiquitin-dependent protein catabolic process
RNA splicing
RNA catabolic process
mRNA catabolic process
proteasomal protein catabolic process
protein targeting
ribosome biogenesis
viral gene expression
rRNA metabolic process
RNA splicing, via transesterification reactions
RNA splicing, via transesterification reactions with bulged adenosine as nucleophile
mRNA splicing, via spliceosome
ncRNA processing
rRNA processing
regulation of mitotic cell cycle phase transition
regulation of cell cycle phase transition
viral transcription
RNA localization
process utilizing autophagic mechanism
autophagy
protein polyubiquitination
macroautophagy
protein folding
nuclear-transcribed mRNA catabolic process
regulation of mRNA metabolic process
ncRNA transcription
DNA replication
nuclear transport
translational initiation
establishment of RNA localization
Go term
0.02
0.03
0.04
0.05
0.06
GeneRatio
$-\log_{10}$(pvalue)
20.0
17.5
15.0
12.5
Count
60
80
100
120
(b)
Biological Process GO Enrichment
proteasome-mediated ubiquitin-dependent protein catabolic process
proteasomal protein catabolic process
mRNA catabolic process
RNA catabolic process
ncRNA processing
RNA splicing, via transesterification reactions
RNA splicing
RNA splicing, via transesterification reactions with bulged adenosine as nucleophile
mRNA splicing, via spliceosome
ribosome biogenesis
protein targeting
translational initiation
nuclear-transcribed mRNA catabolic process
regulation of mRNA metabolic process
RNA localization
viral gene expression
regulation of mitotic cell cycle phase transition
mitochondrial gene expression
regulation of cell cycle phase transition
rRNA processing
cell cycle G2/M phase transition
rRNA metabolic process
G2/M transition of mitotic cell cycle
nuclear-transcribed mRNA catabolic process, nonsense-mediated decay
process utilizing autophagic mechanism
autophagy
mitochondrial translation
viral transcription
macroautophagy
protein-containing complex disassembly
Go term
0.02
0.03
0.04
0.05
GeneRatio
Count
80
120
160
200
$-\log_{10}$(pvalue)
30
25
20

Figure S18

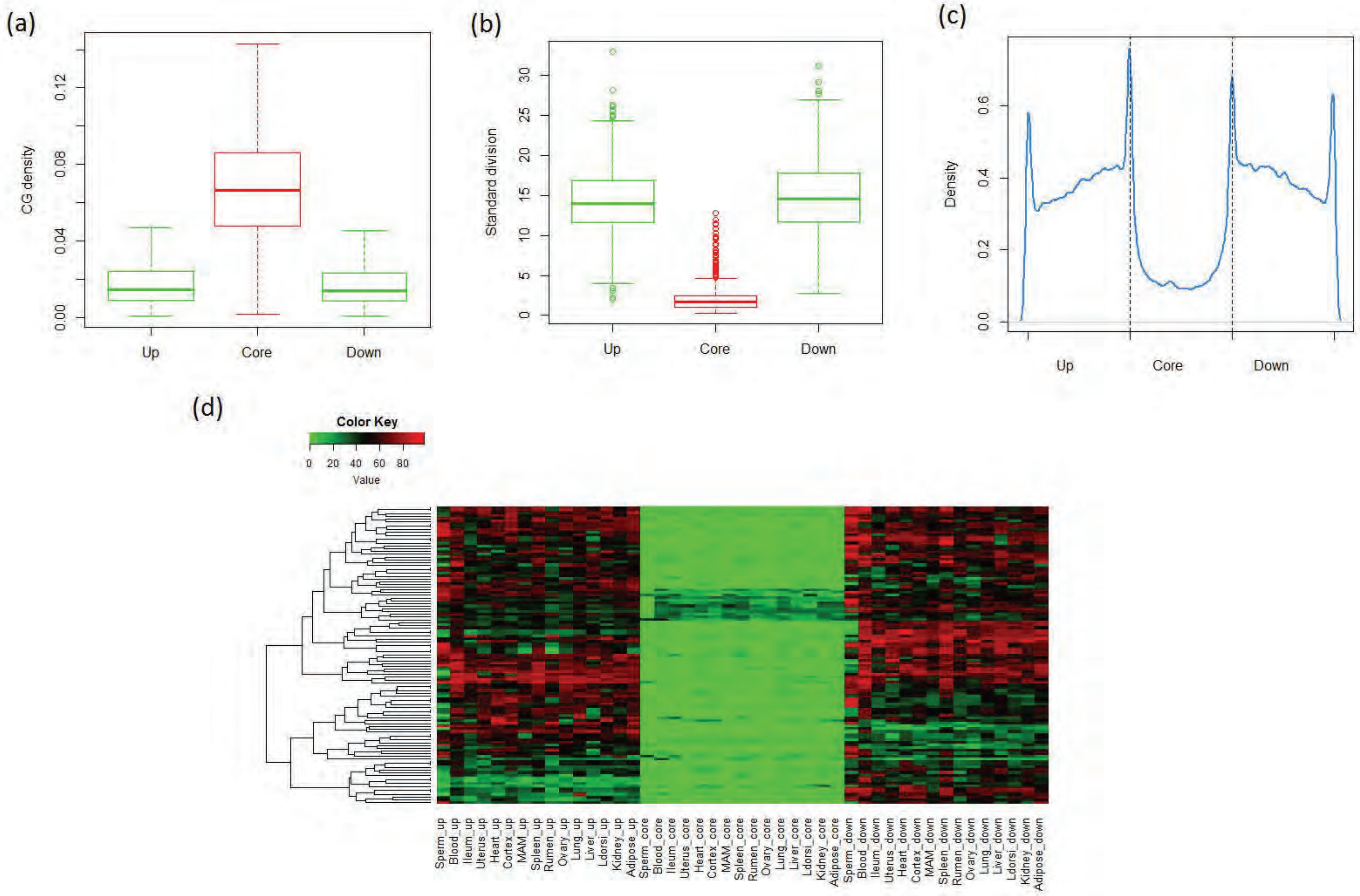
(a)
CG density
0.00
0.04
0.08
0.12
Up
Core
Down
(b)
Standard division
0
5
10
15
20
25
30
Up
Core
Down
(c)
Density
0.0
0.2
0.4
0.6
Up
Core
Down
(d)
Color Key
0
20
40
60
80
Value
Sperm_up
Blood_up
Ileum_up
Uterus_up
Heart_up
Cortex_up
MAM_up
Spleen_up
Rumen_up
Ovary_up
Lung_up
Liver_up
Ldorsi_up
Kidney_up
Adipose_up
Sperm_core
Blood_core
Ileum_core
Uterus_core
Heart_core
Cortex_core
MAM_core
Spleen_core
Rumen_core
Ovary_core
Lung_core
Liver_core
Ldorsi_core
Kidney_core
Adipose_core
Sperm_down
Blood_down
Ileum_down
Uterus_down
Heart_down
Cortex_down
MAM_down
Spleen_down
Rumen_down
Ovary_down
Lung_down
Liver_down
Ldorsi_down
Kidney_down
Adipose_down

Figure S19

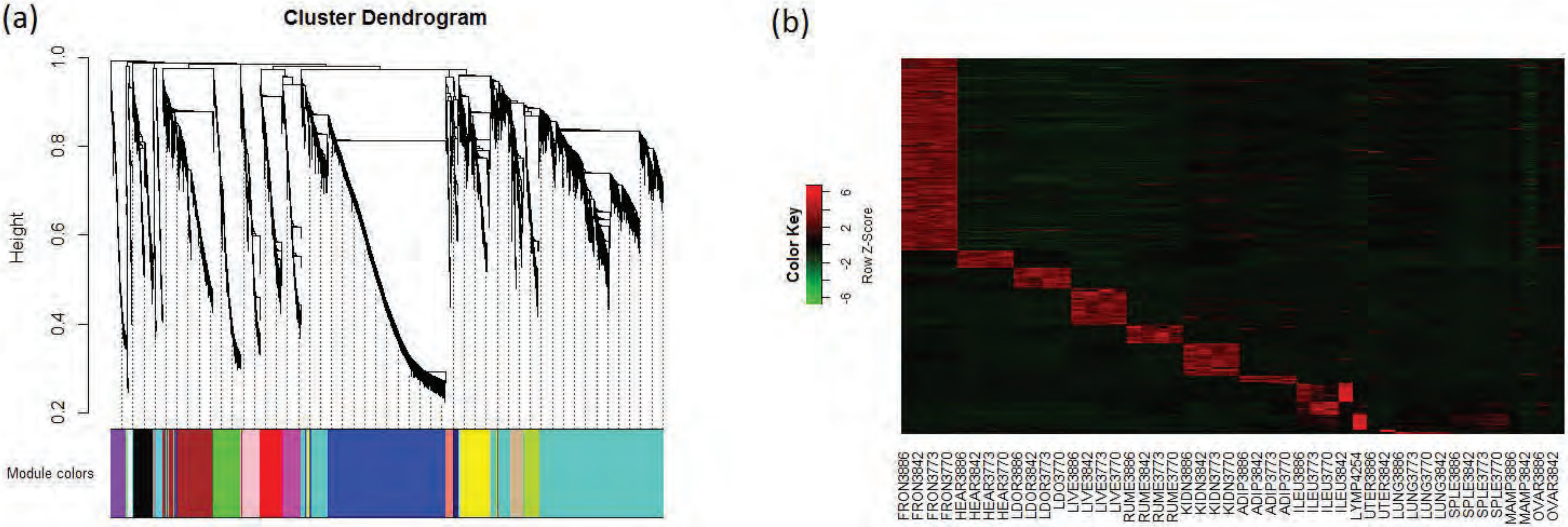
(a)
Cluster Dendrogram
Height
1.0
0.8
0.6
0.4
0.2
Module colors
(b)
Color Key
Row Z-Score
6
2
-2
-6
FRON3886
FRON3842
FRON3773
FRON3770
HEAR3886
HEAR3842
HEAR3773
HEAR3770
LDOR3886
LDOR3842
LDOR3773
LDO3770
LIVE3886
LIVE3842
LIVE3773
LIVE3770
RUME3886
RUME3842
RUME3773
RUME3770
KIDN3886
KIDN3842
KIDN3773
KIDN3770
ADIP3886
ADIP3842
ADIP3773
ADIP3770
ILEU3886
ILEU3773
ILEU3770
ILEU3842
LYMP4254
UTER3886
UTER3842
LUNG3886
LUNG3773
LUNG3770
LUNG3842
SPLE3886
SPLE3842
SPLE3773
SPLE3770
MAMP3886
MAMP3842
OVAR3886
OVAR3842

Figure S20

(a)

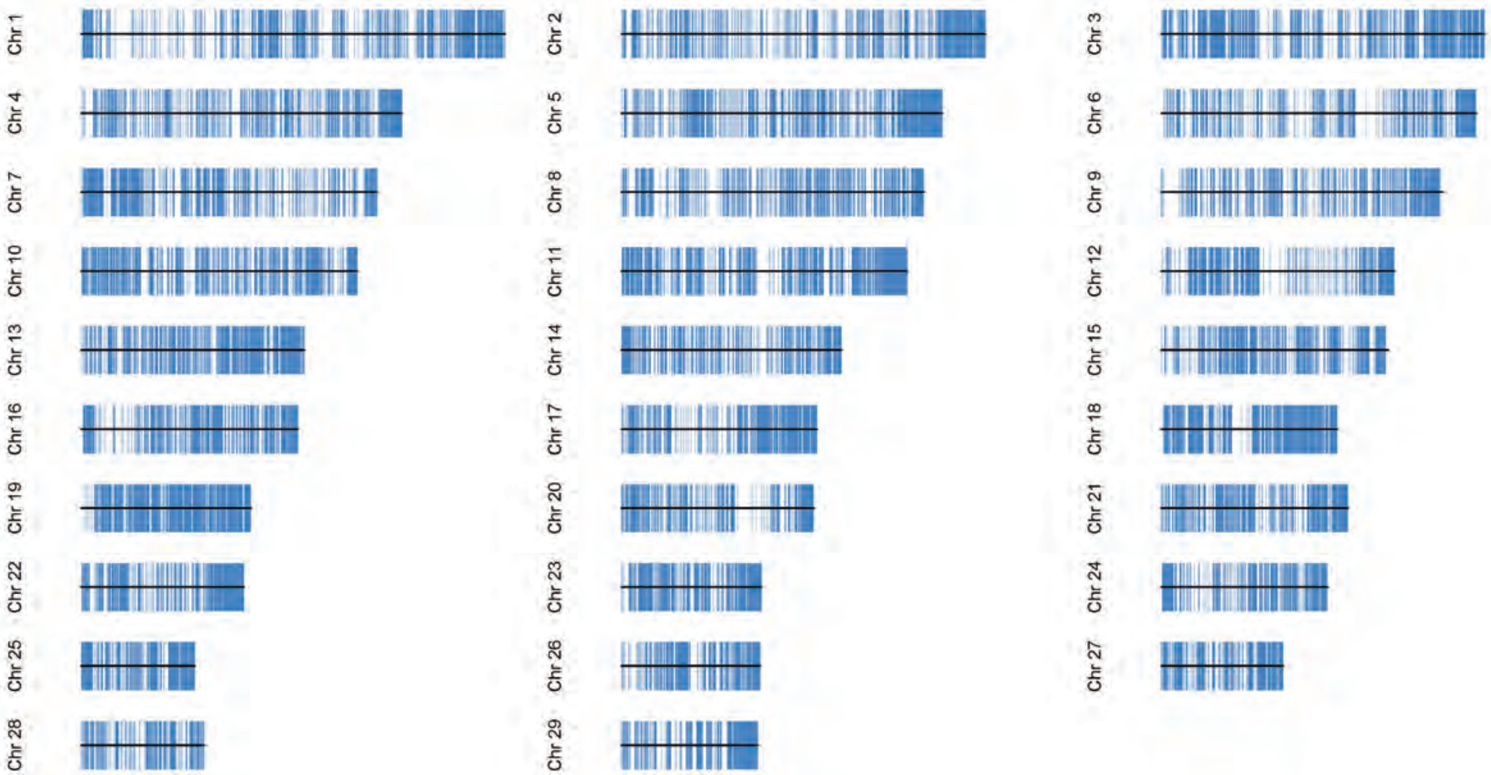


(b)

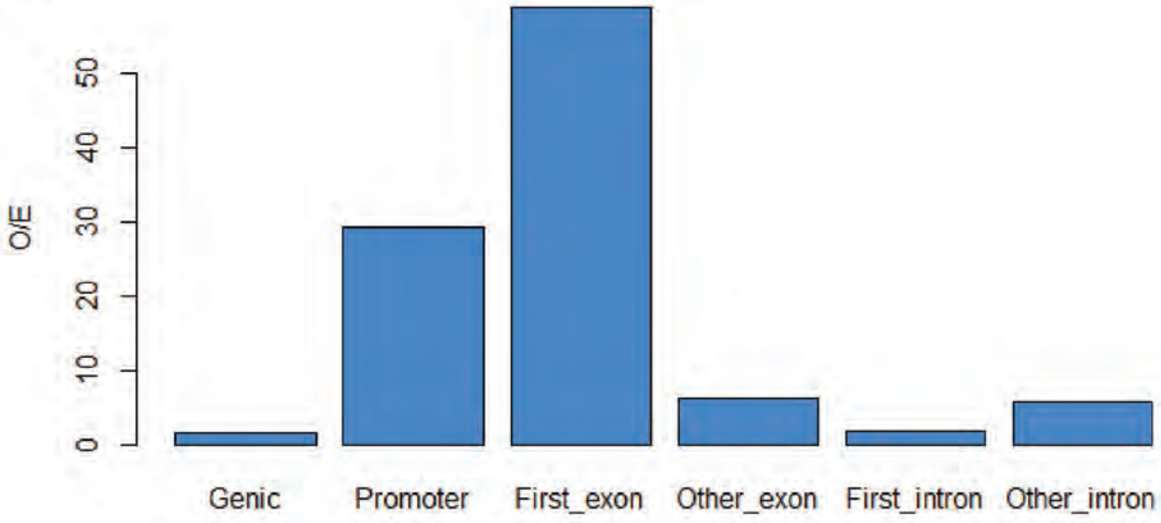


Figure S21

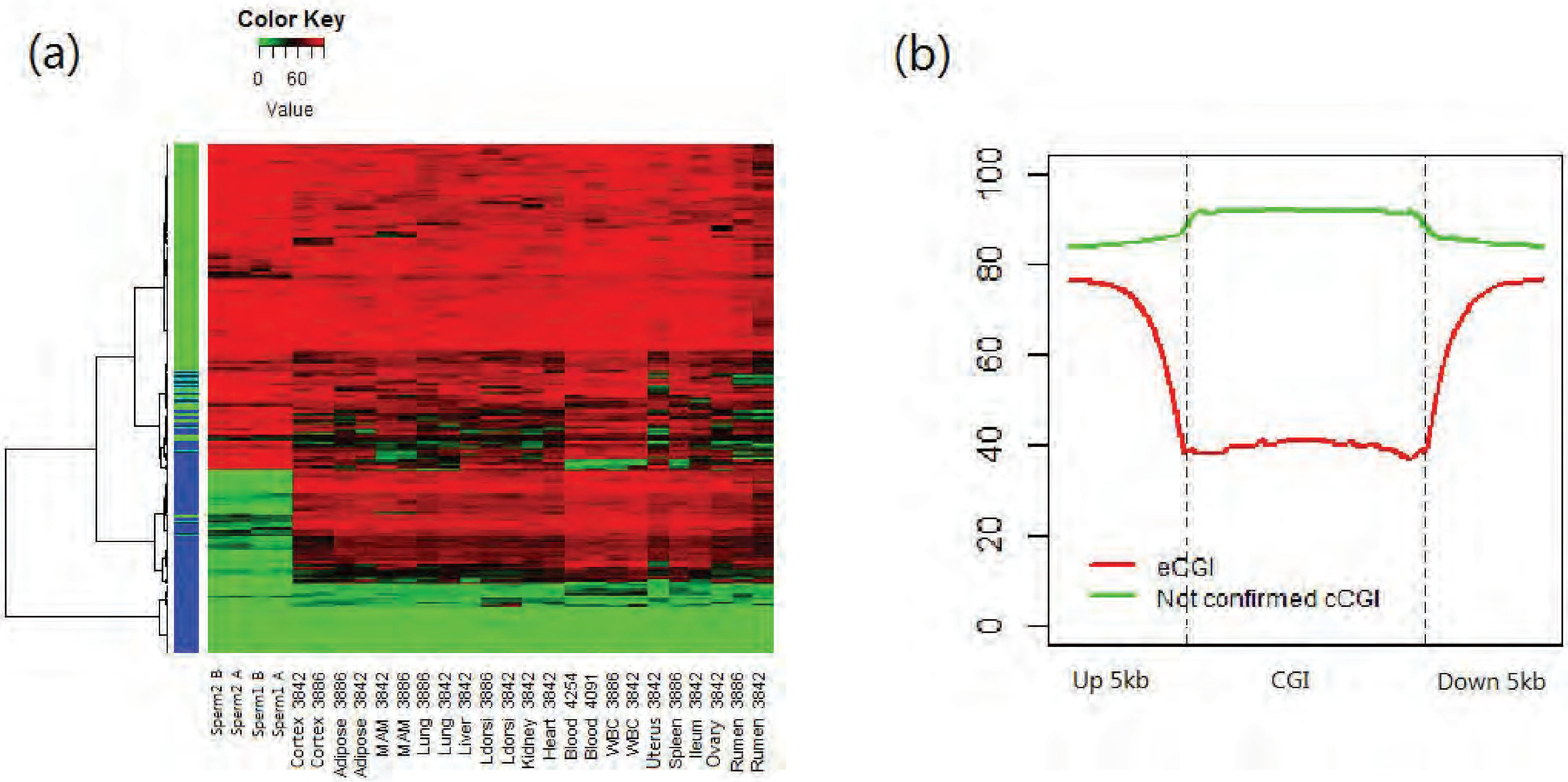

(a)
Color Key
0 60
Value
Sperm2 B
Sperm2 A
Sperm1 B
Sperm1 A
Cortex 3842
Cortex 3886
Adipose 3886
Adipose 3842
MAM 3842
MAM 3886
Lung 3886
Lung 3842
Liver 3842
Ldorsi 3886
Ldorsi 3842
Kidney 3842
Heart 3842
Blood 4254
Blood 4091
WBC 3886
WBC 3842
Uterus 3842
Spleen 3886
Ileum 3842
Ovary 3842
Rumen 3886
Rumen 3842
(b)
100
80
60
40
20
0
eCGI
Not confirmed cCGI
Up 5kb
CGI
Down 5kb


Figure S22

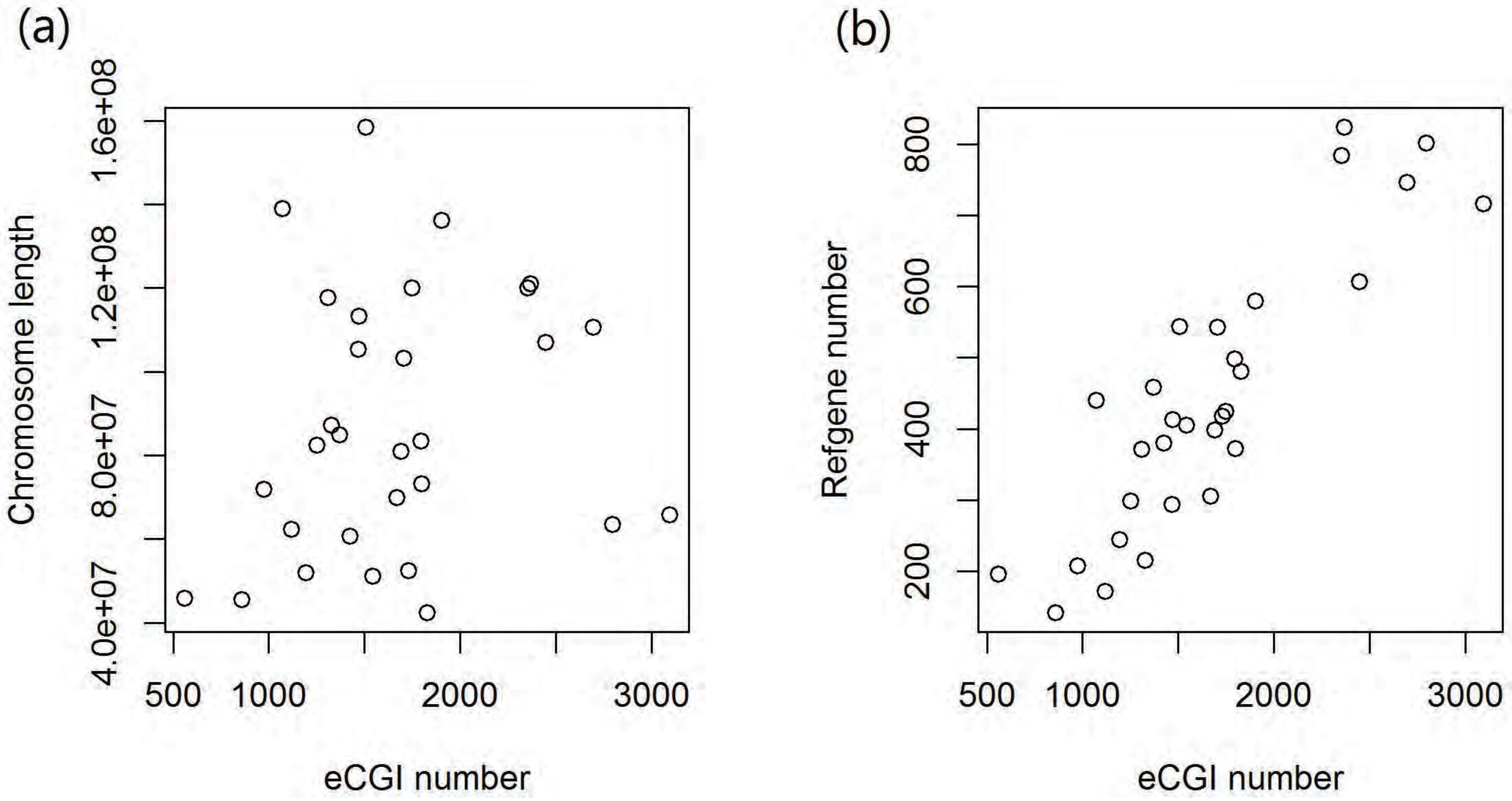
(a)
Chromosome length
4.0e+07
8.0e+07
1.2e+08
1.6e+08
500
1000
2000
3000
eCGI number
(b)
Refgene number
200
400
600
800
500
1000
2000
3000
eCGI number

Figure S23

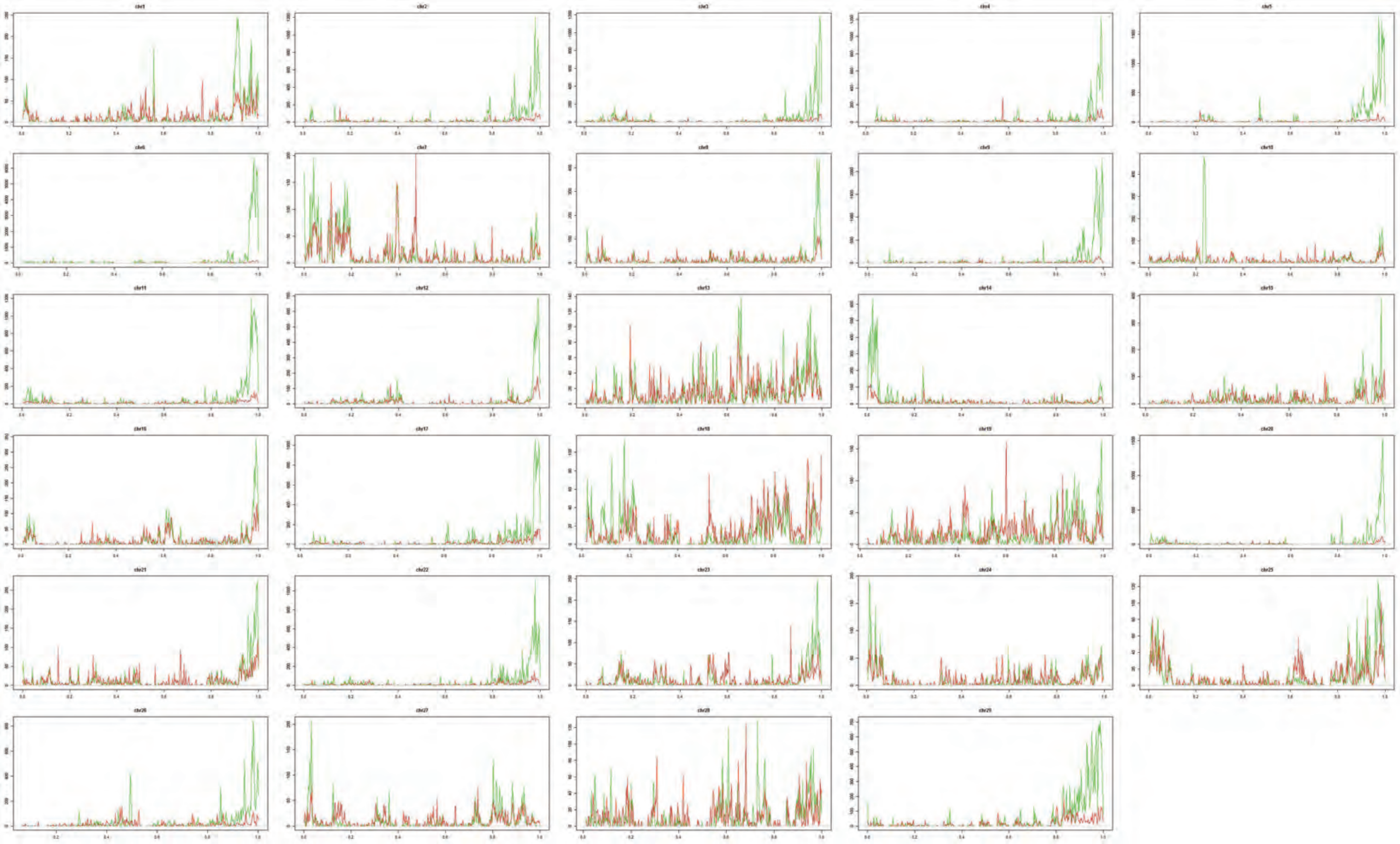

Figure S24

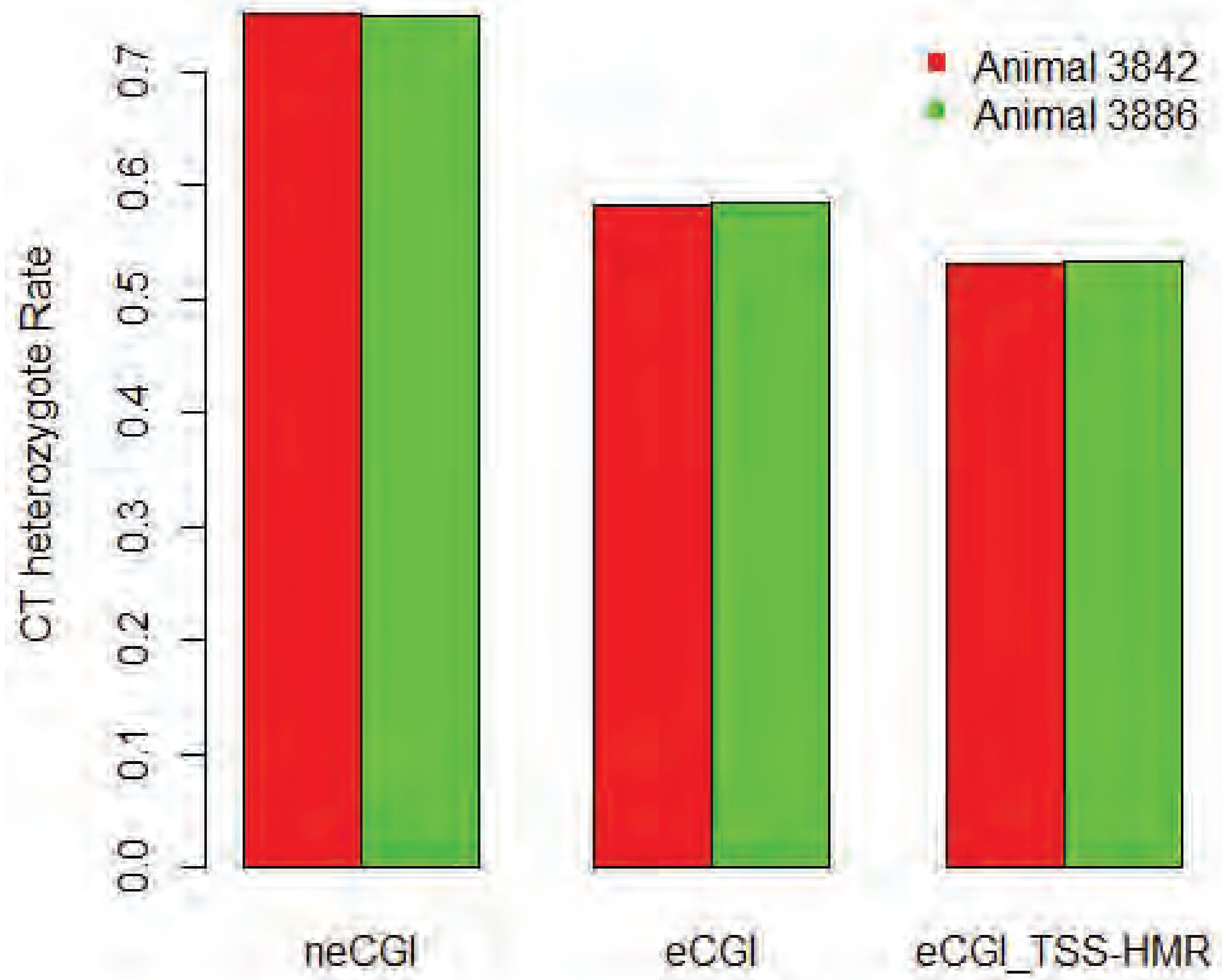
CT heterozygote Rate
0.0
0.1
0.2
0.3
0.4
0.5
0.6
0.7
Animal 3842
Animal 3886
neCGI
eCGI
eCGI_TSS-HMR

Figure S25

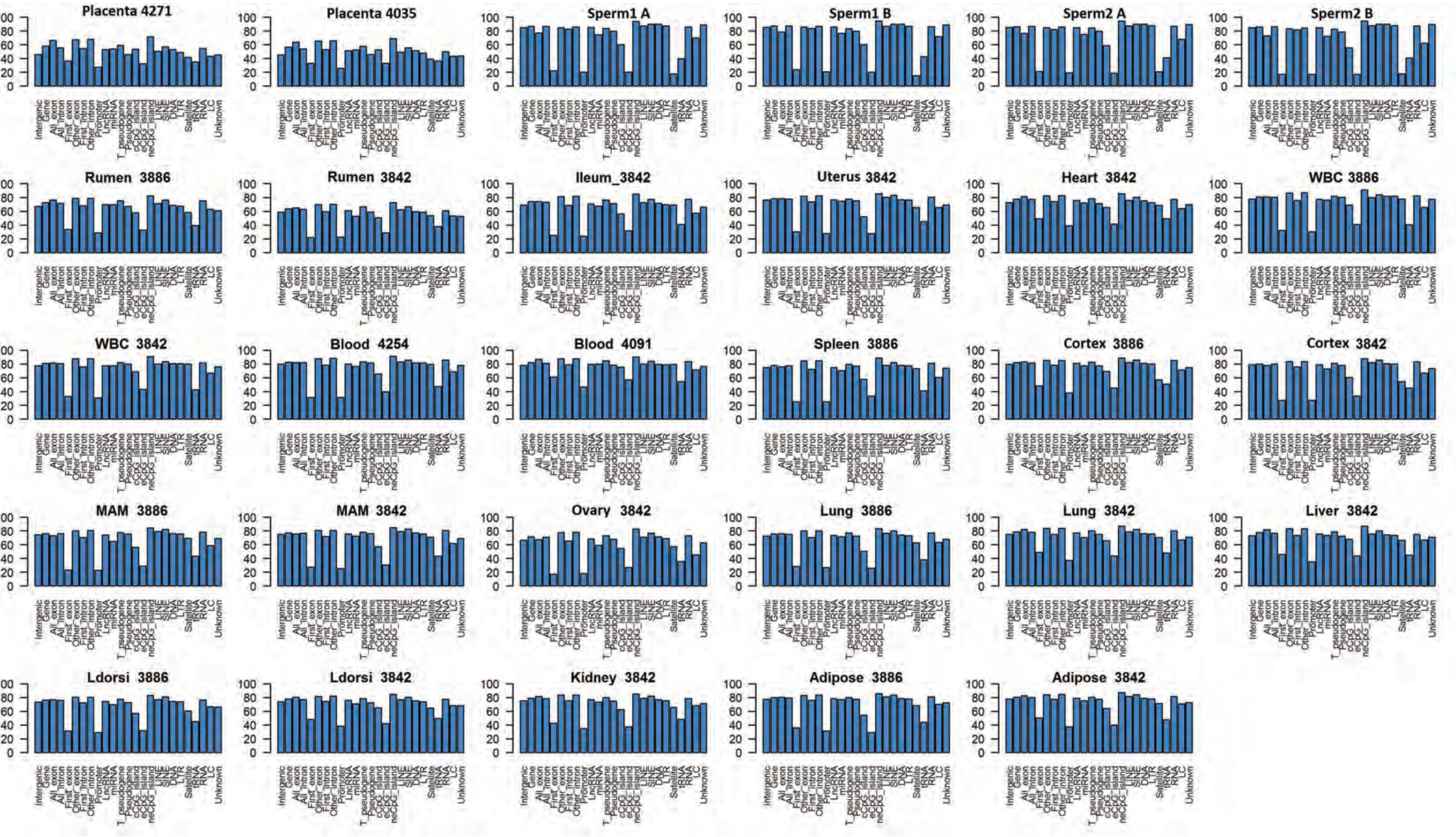
Placenta 4271
Placenta 4035
Sperm1 A
Sperm1 B
Sperm2 A
Sperm2 B
Rumen 3886
Rumen 3842
Ileum_3842
Uterus 3842
Heart 3842
WBC 3886
WBC 3842
Blood 4254
Blood 4091
Spleen 3886
Cortex 3886
Cortex 3842
MAM 3886
MAM 3842
Ovary 3842
Lung 3886
Lung 3842
Liver 3842
Ldorsi 3886
Ldorsi 3842
Kidney 3842
Adipose 3886
Adipose 3842
0
20
40
60
80
100
Intergenic
Gene
All_exon
All_intron
First_exon
Other_exon
First_intron
Other_intron
Promoter
LncRNA
miRNA
T_pseudogene
Pseudogene
cCpG_island
eCpG_island
neCpG_island
SINE
LINE
DNA
LTR
Satellite
tRNA
rRNA
LC
Unknown

Figure S26

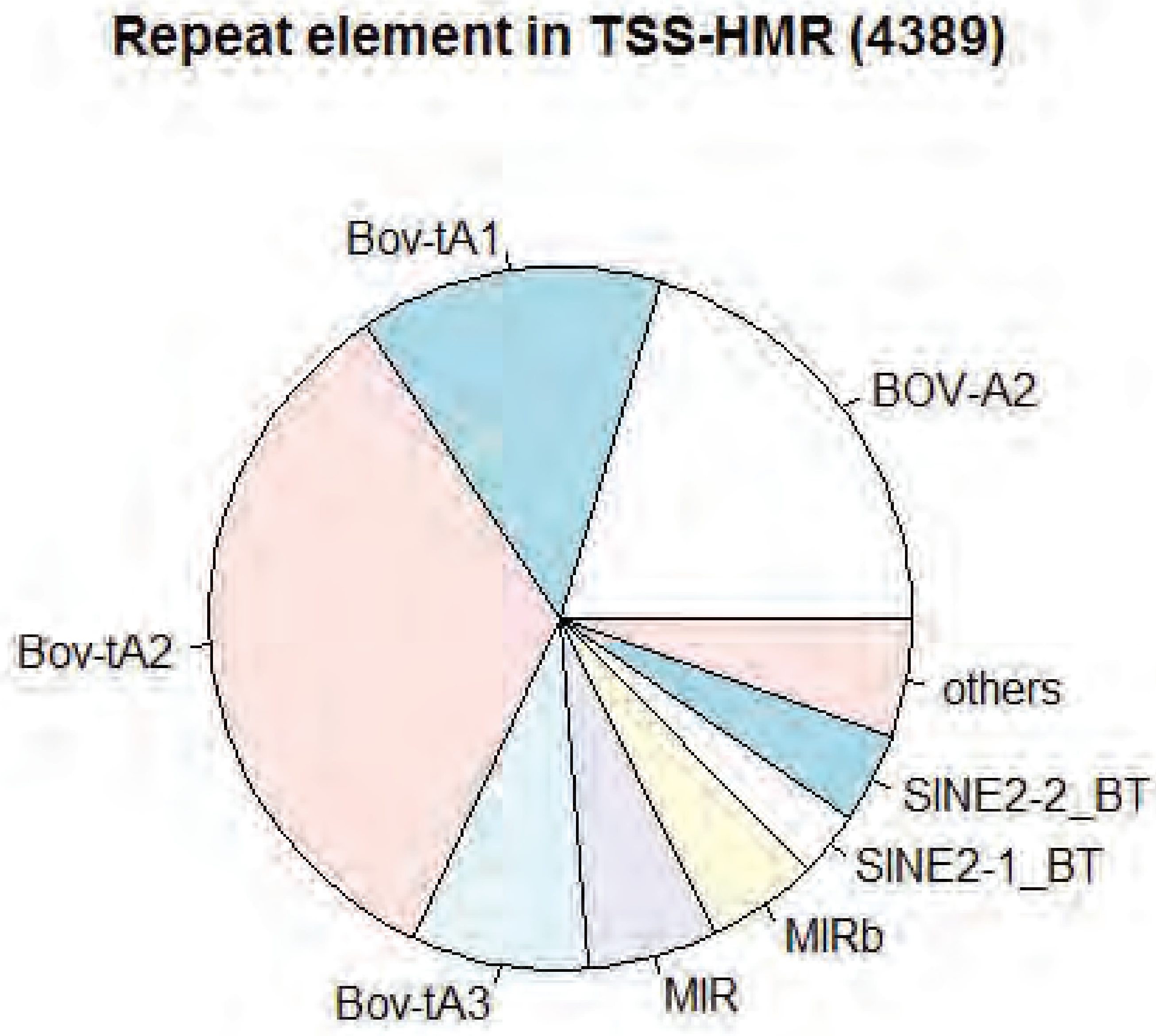
Repeat element in TSS-HMR (4389)
Bov-tA1
BOV-A2
Bov-tA2
others
SINE2-2_BT
SINE2-1_BT
MIRb
MIR
Bov-tA3

Figure S27
